# Quantifying dynamic mechanisms of auto-regulation in *Escherichia coli* with synthetic promoter in response to varying external phosphate levels

**DOI:** 10.1038/s41598-018-38223-w

**Published:** 2019-02-14

**Authors:** Cansu Uluşeker, Jesús Torres-Bacete, José L. García, Martin M. Hanczyc, Juan Nogales, Ozan Kahramanoğulları

**Affiliations:** 1 University of Trento, Centre for Integrative Biology, Trento, 38123 Italy; 2 The Microsoft Research – University of Trento Centre for Computational and Systems Biology, Rovereto, 38068 Italy; 3 Centro Nacional de Biotecnología (CNB-CSIC), Systems Biology Department, Madrid, 28049 Spain; 4 Centro de Investigaciones Biológicas (CIB-CSIC), Microbial and Plant Biotechnology Department, Madrid, 28040 Spain; 5 Institute for Integrative Systems Biology (I2Sysbio-CSIC-UV), Applied Systems Biology and Synthetic Biology Department, Paterna, 46980 Spain; 6 Chemical and Biological Engineering, University of New Mexico, Albuquerque, NM, 87131 USA; 7 University of Trento, Department of Mathematics, Trento, 38123 Italy

## Abstract

*Escherichia coli* have developed one of the most efficient regulatory response mechanisms to phosphate starvation. The machinery involves a cascade with a two-component system (TCS) that relays the external signal to the genetic circuit, resulting in a feedback response. Achieving a quantitative understanding of this system has implications in synthetic biology and biotechnology, for example, in applications for wastewater treatment. To this aim, we present a computational model and experimental results with a detailed description of the TCS, consisting of PhoR and PhoB, together with the mechanisms of gene expression. The model is parameterised within the feasible range, and fitted to the dynamic response of our experimental data on PhoB as well as PhoA, the product of this network that is used in alkaline phosphatase production. Deterministic and stochastic simulations with our model predict the regulation dynamics in higher external phosphate concentrations while reproducing the experimental observations. In a cycle of simulations and experimental verification, our model predicts and explores phenotypes with various synthetic promoter designs that can optimise the inorganic phosphate intake in *E*. *coli*. Sensitivity analysis demonstrates that the Pho-controlled genes have a significant influence over the phosphate response. Together with experimental findings, our model should thus provide insights for the investigations on engineering new sensors and regulators for living technologies.

## Introduction

Mechanisms of inorganic phosphate intake within the context of cellular processes is a topic of extensive research effort, also due to potential applications in enhanced biological phosphorus removal (EBPR) from wastewater. Phosphorous, which is one of the major causes of water quality problems, occurs in wastewater almost solely in the form of phosphates such as inorganic phosphate (*P*_*i*_). Microorganisms, which are key players in bioremediation, have potential to treat large amounts of the pollutants and hold promise for renewable sources^1^. An in-depth understanding of the mechanisms controlling such processes should pave the way for metabolic engineering to lead to improvements in wastewater treatment.

The physiological characteristics of *P*_*i*_ transport in *E*. *coli* have been extensively studied^2–6^. Nonetheless, the regulatory interactions that control the *P*_*i*_ transport are complex, and they involve two major phosphate transport systems, depending on external *P*_*i*_ levels. The low affinity phosphate inorganic transport (Pit) system depends on the proton motive force; it is a coupled transporter of two different ions through the membrane^2,7^. The phosphate specific transport (Pst) system, on the other hand, is *P*_*i*_-repressible, and it is induced when the external *P*_*i*_ concentration is depleted^8–10^.

Mechanistically, *P*_*i*_ signalling associated with the Pst system in *E*. *coli*, is a negative process, that is, excessive *P*_*i*_ is required for turning the system off. Activation is the default state and occurs in conditions of *P*_*i*_ limitation^2,8,11^. Signal transduction by environmental *P*_*i*_ requires seven proteins, which are thought to interact in a membrane associated signalling complex. These *P*_*i*_ signalling proteins include^2,8^.

- Four components of the ABC transporter Pst (PstSCAB), which consist of an extracellular binding protein (PstS), two transmembrane proteins (PstC, PstA) that form the transmembrane domain (TMD), and a dimer of cytosolic peripheral proteins (PstB), i.e., the nucleotide-binding domain;
- Two that are members of the large family of two component systems (TCS) that perform both positive and negative regulation, a sensor histidine kinase PhoR and a transcriptional response regulator PhoB;
- The chaperone-like PhoR/PhoB inhibitory protein PhoU.

**Figure 1:**
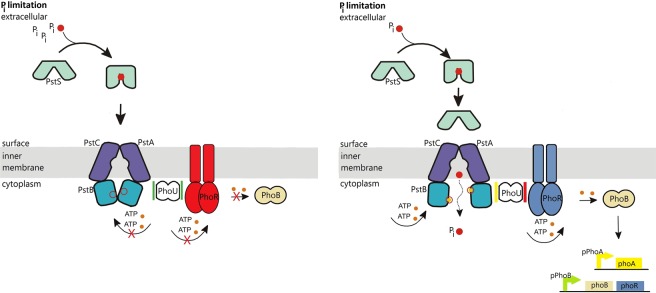
The transmembrane signal transduction due to external *P*_*i*_ levels and the Pho regulon activity when extracellular inorganic phosphate (*P*_*i*_) is in excess (left) and when it is depleted (right). The ABC transporter consists of the extracellular domain PstS, transmembrane domains PstA and PstC, and intracellular domains PstB (see the main text for the description). *Left:* when external *P*_*i*_ is in excess, the ABC transporter activity is limited as the cell does not consume ATP for the *P*_*i*_ transport. According to the current model in the literature, PhoR assesses *P*_*i*_ availability by monitoring the activity of Pst transporter, and relays the signal from PstB via PhoU to PhoR. When the *P*_*i*_ influx is increased, PhoU stabilises PhoR, which is depicted with the green bars around PhoU. This prevents PhoR dimers from autphosphorylating (red). Consequently, the tanscription factor PhoB does not become phosphorylated by PhoR. *Right:* Due to the ABC transporter activity, the external *P*_*i*_ binds to the PstS component of the ABC transporter. It is then translocated to the inner membrane domain of the transporter through PstC and PstA. Following this, PstB changes its conformation by consuming ATP. *P*_*i*_ is internalised and released to the cytosol. When the *P*_*i*_ influx through the ABC transporter decreases, PhoU does not stabilise PhoR, which is depicted with the red bar next to PhoU. As a result of this, PhoR becomes free to perform its auto-kinase-phosphotransferase activity, whereby it phosphorylates PhoB. Phosphorylated PhoB then forms a dimer to act as a transcription factor for the operons, resulting in PhoA, PhoB and PhoR expression.

Figure 1 displays a schematic representation of the system in the starvation condition. When *P*_*i*_ is limited in quantity outside the cell, PstS binds to the *P*_*i*_ following its diffusion to the cell surface^2,8,12^. The transmembrane domain of the ABC transporter, that is, PstC and PstA are integral membrane proteins that span the entirety of the membrane. They regulate the translocation of *P*_*i*_ from PstS to the inner membrane^2,3,8,12^. *P*_*i*_ intake happens with the conformational changes in the PstB as a result of ATP binding, also known as ATP-switch model. This way, the ABC transporter provides the required increase in the amount of phosphate in the cell^2,8^.

The low activity of the ABC transporter PstSCAB causes, in a number of steps, the autophosphorylation of the sensor kinase PhoR, which relays the signal to the transcription factor PhoB. The current evidence suggests that PhoR and eventually PhoB assess *P*_*i*_ availability by monitoring the activity of Pst transporter via PhoU^11,12^. In mechanistic terms, when there is sufficient *P*_*i*_ flux, PhoU stabilizes PhoR. The resulting stable conformation prevents PhoR from auto-phosphorylation. This, in return, prevents the down-stream signalling that would otherwise result in the expression of the genes that feedback to the Pst system for further *P*_*i*_ intake. In fact, when PhoU is deleted, PstB does not only continue to spend ATP and transport *P*_*i*_, but PhoR acts as a constitutive PhoB kinase, leading to high expression of the Pho regulon genes^2,8^, and thereby, activating the expression of the Pst system. On the other hand, when external *P*_*i*_ is limited, PhoU does not stabilise PhoR. Consequently, PhoR is freed to bind phosphoryl groups and passes to the active state. Active PhoR phosphorylates PhoB. Phosphorylated PhoB then acts as a transcription factor for the operon.

All experimental evidence indicates that Pho regulon is controlled by external phosphate limitation rather than internal^3^. When the surrounding environment has abundant phosphate, *E*. *coli* uses as few resources as possible to facilitate the phosphate intake. However, when *P*_*i*_ becomes low outside the cell, it turns into a growth limiting factor and the cell spends energy to up-regulate the expression of target genes that are used to acquire phosphate. Previous studies have shown that the TCS plays a central role in sensing the *P*_*i*_ level in the environment and regulating the expression of genes that are directly involved in the intake^2,12^.

Although much is known about the molecular aspects of this signal transduction pathway, a comprehensive and structured mechanistic model of the Pho regulon is currently unavailable^3^. Here, we investigate the relationship between *P*_*i*_-starvation, the TCS signalling, and promoter activity by using a combination of wet lab experiments and dynamic modelling. This work introduces the proteins involved in phosphate starvation response mechanism. Our model describes the phosphate starvation response at the genetic level by considering all system variables that affect the phosphate starvation response. We consider components where phosphate starvation response of TCS model is described in terms of their interactions. The model is composed of a set of ordinary differential equations and the corresponding stochastic model that are derived from a chemical reaction network. The model provides a quantitative description of how different processes interact to form a positively-regulated biological control system. The mechanism is based on an *E*. *coli P*_*i*_-starvation signalling system. Our model displays how specific TCS proteins work together to provide gene expression and increase in *P*_*i*_ intake, and provides a set up for identifying a wide family of promoter mechanisms that potentially have synthetic applications.

In recent years, significant advances have been made in understanding the role and the structure of TCS and signalling mechanisms. It is known that most bacteria species have more than 10 different TCS^13^. Moreover, in *E*. *coli*, 30 sensor kinases and 32 response regulators have been found^14^. Although there is large qualitative knowledge, especially for *E*. *coli*, quantitative research is still scarce. A model for PhoR/PhoB signal transduction was set up by Van Dien and Keasling earlier^3^, whereby the authors have determined a model for the induction of PhoA. Models for other signal transduction systems in E. coli are described by Wong *et al*., Fisher *et al*., and Koh *et al*.^15–17^. Recently, research groups have only just begun to use modelling in combination with experimental data to analyse the TCS especially PhoR and PhoB^4,18^. Gao *et al*. built quantitative analyses of PhoR and PhoB protein concentrations and activities. They showed that the experimental data and protein expression levels of TCSs matched. Recently, Gao and Stock *et al*.^19^ built a quantitative analyses of TCS switch off mechanism. However, to the best of our knowledge, there has been no focus on developing genetically encoded signal transduction pathways leading to transcription factors.

In this work, we have developed an approach combining wet-lab experiments and modelling to show that rates of histidine kinase and promoter activities can be used to tune TCS detection thresholds. We have described the steps in the design of a synthetic biological system based on the use of TCS. This should constitute a contribution to the research on genetically modified bacteria that detect environmental changes and respond to higher inorganic phosphate levels. In order to better understand the quantitative analyses of protein concentrations in response to modifications and activities in the environment, we have focused on the activation response of the TCS components PhoB and PhoR. Our model should thus help to better understand the dynamic behaviour of system activation, and to quantitatively evaluate the role of phosphatase activity under varying external *P*_*i*_ conditions.

The model includes TCS members and activation of the Pho regulon promoters pPhoB and pPhoA. Experimental data is used to fit the parameters to the feasible physiological range given in the literature, and to determine the relative sensitivity to the parameters. The simulations with our model provide a dynamic description of the mechanisms. With a combination of wet-lab experiments and computer simulations, we use our model to quantify dynamic mechanisms of auto-regulation in *E*. *coli* in response to varying external phosphate levels, and explore and verify emerging phenotypes with synthetic promoters. Simulations with the model do not only reproduce our experimental measurements, but also predict phenotypes with various synthetic promoter designs that can optimise the *P*_*i*_ intake in *E*. *coli*. Sensitivity analysis on the parameters demonstrate the influence on the expression of Pho-controlled genes and the gain of the system under variations in transcription efficiency in response to external phosphate concentration. The model can thus serve as a virtual lab, and can be used to test various promoter designs for enhanced *P*_*i*_ intake in biotechnology applications for phosphate sequestration.

## Results

### Phosphate intake at starvation requires rapid activation of PhoB dimers

**Figure 2:**
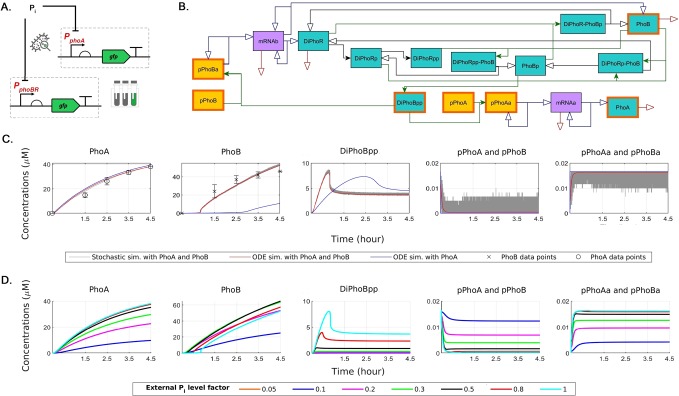
Schematic representation of the experimental data and the control model, and its dynamics in response to varying external *P*_*i*_ concentrations. (**A**) The data for the PhoA and PhoB expression are obtained using PCR amplified DNA from *E*. *coli* MG1655 genome and transcriptionally fused to the translational coupler BCD2 and the fluorescent *ms-fgfp* gene. (**B**) The control model has been obtained from the experimental data and the chemical reaction network (CRN) described in Methods by applying a fitting procedure with the physiological ranges obtained from the literature in Table 1, and verified by sensitivity analysis. The deterministic ODE and stochastic simulations are performed by applying the standard translation from CRNs based on stoichiometry. The blue color denotes the proteins, orange denotes the promoters in their active and inactive forms, and purple denotes the mRNA molecules. Filled arrowheads denote the reversible reactions. The red arrows denote degradation reactions, the green arrows denote complexations, the black arrows denote phosphorylation and dephosphorylation, and the blue arrows denote the transcription and translation reactions. The model species that are plotted in C and D are distinguished with frames. (**C**) The dynamics of the highlighted species of the control model in panel B as a result of the fitting procedure together with the experimental data, as described in Methods, are plotted. In the first of the two steps, the blue curves are obtained by using only the experimental values of PhoA levels. In the second step, that delivers the control model, the red curves are obtained by using the experimental values of both PhoA and PhoB levels. Inclusion of the PhoB data highlights the contribution of the feedback mechanism to the response dynamics, which is otherwise not represented. The stochastic dynamics, plotted in grey, display the fluctuations in the control model that are due to small molecule numbers and are not observable in the deterministic simulations. (**D**) The response of the control model to variations in the external *P*_*i*_ levels, which are represented as fold change factors, applied to the autophosphorylation propensities of PhoR. A higher external *P*_*i*_ concentration corresponds to a smaller factor and vice versa.

Figure 2 provides a schematic representation of the control model set up with the experimental data and the formal model. The control model is set up with respect to the initial phosphate starvation in accordance with the experimental data and the parametrisation and fitting procedure described in Methods. In first of the two steps, the blue curves in Figs 2C and S2 are obtained by using only the experimental values of PhoA levels. When external *P*_*i*_ is abundant, the Pst system inhibits the activation of TCS, and consequently the *P*_*i*_ intake; PhoA is then expressed at a basal level. However, when *P*_*i*_ is limiting, inhibition of TCS is relieved, resulting in the activation of PhoA transcription. The alterations of PhoA expression can thus be interpreted to an extent as an indicator for the changes in external phosphate level and *P*_*i*_ intake.

In these simulations, in response to external *P*_*i*_ level at 0 *μM*, the system initiates the activation of PhoR, given by the autophosphorylation of both of the monomers in the stable dimer. This results in the subsequent transfer of phosphoryl groups. As a consequence, the response regulator PhoB rapidly becomes active and dimerises to form active transcription factors. The resulting rapid increase in the promoter activity delivers the mRNA transcription, and the consequent experimentally observed levels of PhoA.

At a second step in our analysis, to highlight and contrast the role of PhoB dynamics in the feedback mechanism, we have refined the model to include the experimental data on PhoB expression. The resulting red curves in Figs 2C and S2 are obtained by using the relative experimental levels of both PhoA and PhoB in the fitting procedure. The rates in Table 1 are obtained as a result of this fitting procedure that delivered the control model parameters, whereby we have enforced the displayed physiologically boundaries. The difference in the phenotype between red and blue curves in Fig. 2C should thus highlight the role of the feedback of the PhoB and PhoR expression to the system.

**Table 1:** Reactions and deterministic rates obtained from the physiological ranges.

Reaction Number	Rate Symbol	Fit Value	Literature Value	References
1	r1	25.3658	10–100 *s*^−1^	^5^
1 reverse	r1r	8.1165	$\ll \,10\,{s}^{-1}$	^5^
2	r2	25.3658	10–100 *s*^−1^	^5^
2 reverse	r2r	8.1165	$\ll \,10\,{s}^{-1}$	^5^
3	r3	100	100 *μM*^−1^ *s*^−1^	^48^
3 reverse	r3r	44.9411	N.A.	N.A.
4	r4	21.3718	17–23 *s*^−1^	^5^
5	r5	100	100 *μM*^−1^ *s*^−1^	^48^
5 reverse	r5r	94.9411	N.A.	N.A.
6	r6	21.3718	17–23 *s*^−1^	^5^
7	r7	100	100 *μM*^−1^ *s*^−1^	^48^
7 reverse	r7r	24.9411	N.A.	N.A.
8	r8	100	100 *μM*^−1^ *s*^−1^	^48^
8 reverse	r8r	34.9411	N.A.	N.A.
9	r9	12.95	$\ll \,17\,{s}^{-1}$	^5^
10	r10	10000	10000 *μM*^−1^ *s*^−1^	^6^
10 reverse	r10r	1000	N.A.	^6^
11	r11	10000	10000 *μM*^−1^ *s*^−1^	^6^
11 reverse	11r	1000	N.A.	^6^
12	r12	0.0540	0.0025–0.2 *s*^−1^	^47, 49^
13	r13	0.0302	0.0006–0.05 *s*^−1^	^47, 50^
14	r14	0.130	0.0025–0.2 *s*^−1^	^47, 49^
15	r15	0.035	0.0006–0.05 *s*^−1^	^47, 50^
16	r16	0.0302	0.0006–0.05 *s*^−1^	^47, 50^
17	r17	0.0001	0.000096–0.00079 *s*^−1^	^47, 49, 50^
18	r18	0.0001	0.000096–0.00079 *s*^−1^	^47, 49, 50^
19	r19	0.0001	0.000096–0.00079 *s*^−1^	^47, 49, 50^
20	r20	0.0055	0.0055 *s*^−1^	^47^
21	r21	0.0055	0.0055 *s*^−1^	^47^

We have performed a large scale analysis of the system dynamics of the control model in terms of the fitted parameter values within a broader range. We have first analysed the broader effect of the TCS disassociation rates (r3r, r5r, r7r, r8r) on the dynamics and the PhoB transcription and translation parameters that deliver the expression of PhoB (r14, r15), which is a transcription factor. The cumulative output of different simulations with fold changes that cover the physiological intervals for r14 and r15 as well as a broad range for r3r, r5r, r7r, and r8r are depicted in Figs S3, S4, S5, S6, S7 and S8, respectively.

In these simulations, the dissociation rates of PhoR and PhoB, that is, r3r and r5r, have transient effects on the steady state concentrations of the complexes formed by these molecules. These rates do not modify the PhoB activity or the levels of PhoA and PhoB as shown in Figs S5 and S6. Variations in the dissociation rate of PhoB, given by r7r, affect the activity of PhoB as a transcription factor, however this has a minor effect on the transcription and translation of PhoB and none on PhoA (Fig. S7). On the other hand, the dissociation rate of the inactive PhoR from the active PhoB, given by r8r, affects the activation of PhoB as a transcription factor in a proportional way, yet its effect on promoter activity is negligible (Fig. S8). As it can be seen from Table 1, there are also other parameters that have an effect on both system dynamics and PhoB activity. Nonetheless, these are not suitable candidates for modification at this first analysis due to their physiologic ranges, and they are discussed below.

As it can be observed in Fig. 2, and supported by the analysis above, the simulations that include the PhoB data for parametrisation result in faster response dynamics, measured in terms of the time required to reach a peak state. This can be explained by the self-feeding role of the TCS, and the resulting increased requirement for the active transcription factors to sustain the experimentally observed protein levels due to their feedback to the network: because PhoR and PhoB are encoded by the same operon, not only PhoB levels, but also PhoR levels increase as a result of the changes in the reaction rates. This causes the cell to increase the sensor histidine kinase levels, resulting in a more immediate response. The response time thus decreases with an increase in the amount of sensor histidine kinase as well as an increase in its activity.

The deterministic ODE simulations lead to observations that describe the average dynamic behaviour of the variable concentrations for the simulated 4.5 hours. To observe the possible fluctuations in the system, we have performed stochastic simulations. This way, we have been able to compare the mean behaviour with the regulatory dynamics that incorporates the noise due to smaller molecular numbers. Figure 2 as well as Figure S2 include a comparison of the deterministic and the stochastic simulations. The stochastic simulations are performed by applying the standard conversion to obtain molecule numbers from the concentrations. The grey fluctuating lines show the stochastic results at a single representative simulation; the simulation shows the expected fluctuations in the model species with smaller numbers such as mRNA molecules as well as the qualitative agreement between the stochastic and the deterministic simulations.

Binding and unbinding of transcription regulators are a primary mechanism for gene regulation, whereby transcription factors operate at a fast time-scale. While the rate of binding of transcription regulators are known in many cells, little is known about how cells can modulate their unbinding for regulation^20^. The unbinding rate of an active transcription factor can thus vary over many orders of magnitude^6,20^. Therefore, in the initial analysis, we have fixed the transcription factor unbinding rates (r10r, r11r) to 1000 *s*^−1^ as shown in Table 1, and analysed the system behaviour with respect to variations.

**Figure 3:**
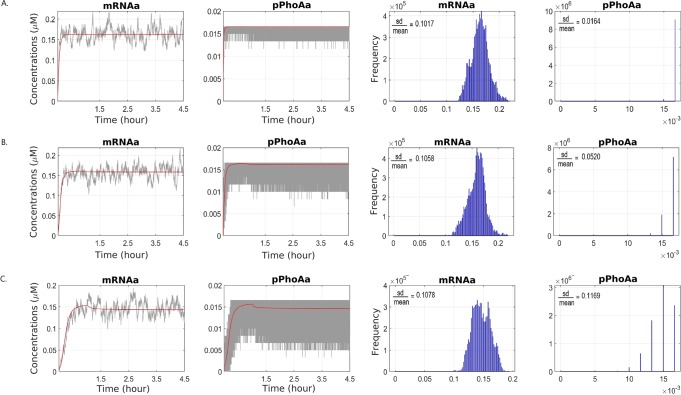
The variations in the mRNA and active promoter levels due to the unbinding rate of the promoter and the active transcription factor. An unbinding rate of 100/*sec* (**A**) results in much less spread in the steady state distributions in comparison to unbinding rates of 1000/*sec* (**B**) and 5000/*sec* (**C**). The variations are quantified as the ratio of the standard deviation and the mean.

To this end, we have experimented in stochastic simulations with different DNA unbinding rates, that is, r10r and r11r, from 100/*s* up to 5000/*s*. In accordance with the common practice, we have used stochasticity to quantify the noise that arises from the binding of a regulatory protein to a promoter^21^. The resulting amplification in oscillations in stochastic simulations, shown in Fig. 3 as well as Figs S9, S10 and S11, are due to the increase in the promoter unbinding rates. In Fig. 3, we have quantified the decrease in noise in the steady state distribution of mRNA and active promoter levels in terms of the ratio of the standard deviation over the mean. In this respect, the deterministic simulations display how DNA binding rates affect the mean behaviour, while the stochastic simulations bring about the loss of coherence due to noise in gene expression. Figure 3B displays the simulations with the rates of the control model. In Fig. 3A, DNA unbinding rate is decreased by an order of magnitude, whereas in Fig. 3C, the effect of a higher DNA unbinding rate of 5000/*s* is depicted. These results indicate that lower unbinding rates, as observed in saturation conditions, are required for stable gene regulation that is not affected by noise. This also reflects how both genetics and noise due to environmental factors can affect the development of targeted pathway interventions for faster *P*_*i*_ accumulation.

The results above show that our control model provides detailed predictions about the complex effects of production pathways of the *P*_*i*_ accumulation system. The simulations are in good agreement with the experimental data and the general concepts described in the literature for the functionality of Pho regulon^2,3^. The control model proposed can thus serve as a virtual lab, which can be used to test and justify the theoretical approaches on the *P*_*i*_ intake system.

### PhoR tunes the *P*_*i*_ intake both up and down

Besides the chemical properties of the proteins in the regulatory system^4^, also the activity of the TCS proteins influence the *P*_*i*_ intake. Moreover, as displayed in Fig. 2C, the feedback mechanism due to the increased expression of sensor histidine kinase PhoR and the response regulator PhoB introduces a speed-up of an hour in comparison to the simulations, where this feedback mechanism is not taken into consideration. This indicates that a faster response in terms of *P*_*i*_ intake is delivered by an increase in the histidine kinase levels as well as the increase of its activity due to signalling.

A notable feature of the *P*_*i*_ response system is that the sensor histidine kinase is bifunctional: it participates in both phosphorylation and dephosphorylation of its cognate response regulator. In this respect, the TCS autoregulatory design is a distinct mechanism from the conventional positive feedback loops. The bifunctional PhoR component is an autokinase with concomitant opposing phosphatase activity^22–24^.

The dual role of PhoR is a mechanism that enhances signal robustness^18,25^. Moreover, it has also been shown that the phosphatase activity in TCS provides a rapid dephosphorylation mechanism that shuts off the system, and thereby restores it to the original state^19^. Such a dynamics can be triggered, for example, by an increase in the external *P*_*i*_ concentration and the consequent decrease in the autophosphorylation activity of PhoR.

We have analysed the effect of the changes in autophosphorylation rates to the system behaviour. Because autophosphrylation becomes possible when the starvation signal prevents PhoU from inhibiting PhoR, the propensity of autophosphorylation depends on the incoming signal, which is a function of the external *P*_*i*_ levels. By decreasing the autophosphorylation propensity by applying various fold changes, we can thus see the effect of an increase in external *P*_*i*_ concentration on the system as depicted in Figs 2D and S12. In these simulations, a decrease in PhoR activity due to increased external *P*_*i*_ concentration results in a proportional decrease in the active PhoB dimers, and a decrease in the promoter activity as well as the PhoR activity.

A complementary realisation of this mechanism is given by the association of PhoB to PhoR, that is, r8. Although the physiological range for this parameter is narrow, as a result of a hypothetical increase in the association rate of PhoB and PhoR, the PhoB concentration stays low for a longer time period and the levels of active PhoB dimers decrease proportionally as displayed in the fold change experiments in Fig. S13.

In our model, we assume that the growth in the cell culture within the considered time interval is negligible. However, due to cell cycle, which has a time scale in the order of an hour, the protein concentrations can be subject to dilution besides the active degradation of the molecules we have considered. To this end, Figs S14, S15 and S16 explore the effect of higher degradation rates due to dilution in growth conditions, (r17, r18, r19, r20, and r21), together with higher external *P*_*i*_ levels, given with a decrease in the rates r1 and r2. As it can be seen in Fig. S12, a decrease in the autophosphorylation rates does not only lower the steady state levels, but also slows down the activation of the transcription factor by preventing the formation of an initial peak in DiPhoBpp levels. A concomitant increase in the degradation and dilution rates delays reaching a steady state. However, this does not drastically alter the eventual active DiPhoBpp concentrations.

### Starvation response can be obtained with synthetic promoters at higher external *P*_*i*_ concentrations

The simulation results in Figs 2D and S12 demonstrate the system’s adaptation to the stimuli due to *P*_*i*_ concentration, whereby the autophosphorylation propensity of PhoR acts as a proxy for the external *P*_*i*_ levels. These simulations predict how changes in the external *P*_*i*_ concentration affect the Pho regulon, and in particular, how the promoter activity decreases with an increase in the external *P*_*i*_ concentration. These results thus confirm that the adaptation of gene expression is clearly dependent on the *P*_*i*_ response stimuli^2,12^. Moreover, the model provides a mechanistic explanation for the interplay between the system components under the conditions of varying external *P*_*i*_ concentrations, which result in variations in the promoter activity.

**Figure 4:**
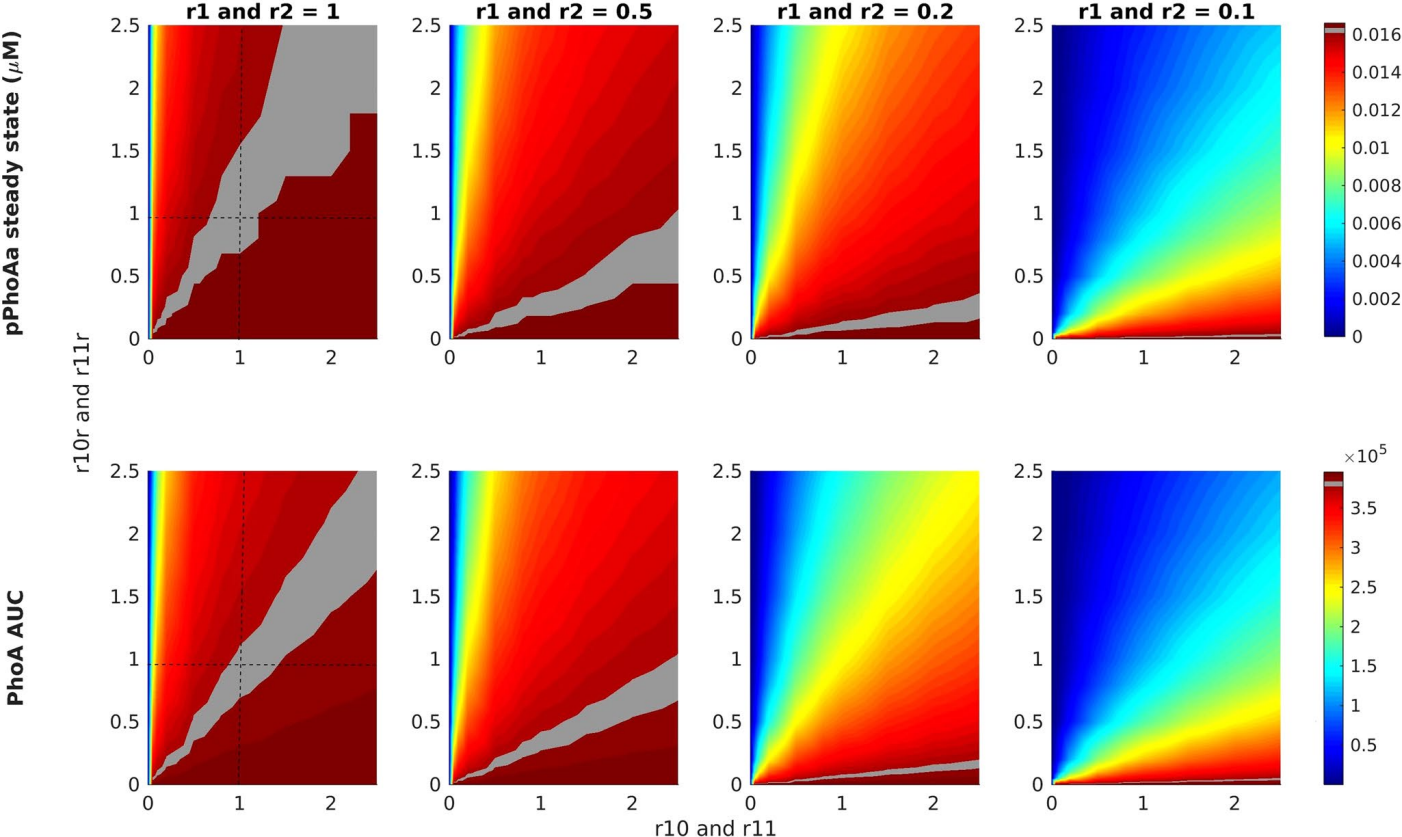
Heatmaps for the activity of various promoter designs for pPhoA and pPhoB, and the resulting PhoA expression (bottom-row) under different external *P*_*i*_ concentration conditions. The heatmaps are ordered decreasingly from left to right according to the external *P*_*i*_ concentration given by the fold changes applied to the PhoR autphosphorylation reactions r1 and r2. The left most column with 1 as the fold change value is the starvation condition with 0 *μM* external *P*_*i*_. Each heatmap scans 100 simulations by applying 10 different fold change values to the promoter binding rates r10 and r11 as well as 10 different fold change values to the promoter unbinding rates r10r and r11r. The upper row displays the resulting steady state levels of the active promoter pPhoAa, whereas the lower row displays the yield of PhoA gene expression measured as the area under the curve (AUC). The intersection of the dashed lines in the left column delivers the experimentally observed regime observed in Fig. 2. The levels of this regime, that display the starvation response, are highlighted in all the heatmaps.

It is well established that the protein production rate is greatly influenced by the specific nucleotide sequence of the promoter^26–28^. In this respect, synthetic biology and genetic engineering methods aim at synthesising promoters with the desired strength. To this end, in order to observe the possible variations in gene expression due to variations in promoter strength, we have performed a class of simulations. The results of these simulations in Fig. 4 display measurements of the steady state levels of PhoA promoter activity (pPhoAa) as well as the PhoA yield of the system as the resulting product in terms of the area under the curve (PhoA AUC).

In these simulations, we have scanned regimes with varying external *P*_*i*_ concentrations simulated by applying fold changes to the rates r1 and r2 as in the simulations in Figs 2D and S12. We have applied a fold change of 1 for the control regime with 0 *μM* external *P*_*i*_, and 0.5, 0.2 and 0.1 for increasing external *P*_*i*_ levels. For each external *P*_*i*_ regime, we have scanned 100 different promoter designs by means of simulations that apply 10 fold change values from 0 to 2.5 with steps of 0.25 to the promoter binding rates r10 and r11 as well as 10 such fold change values to the promoter unbinding rates r10r and r11r. The heatmaps resulting from these 100 simulations are depicted in Fig. 4, where the control values for 1 fold change for binding and unbinding rates are marked by dashed lines.

We have further assessed our analysis to factor for the possible effects of stress response in *P*_*i*_ starvation conditions. As depicted in Fig. S17, *P*_*i*_ limitation induces the general stress response regulated by RpoS, sigma factor^29,30^. Concomitantly, *P*_*i*_ limitation increases the intracellular level of guanosine tetraphosphate (ppGpp), which is known as stringent stress response^12,30,31^. RpoS and ppGpp are thought to maintain a balance between vegetative and stress/starvation states of *E*. *coli*. The ppGpp nucleotide induces RpoS accumulation^32^. The RpoS responds to stress and shifts transcription away from vegetative growth and towards stress resistance^31–34^. Accumulation of ppGpp triggers the stringent response and a radical decrease in ribosome and protein synthesis, even leading to growth arrest^31^. When the level of RpoS is higher, *E*. *coli* is more resistant to stress but grows more slowly under a variety of conditions^32,33^.

It is known that RpoS and ppGpp direct RNA polymerase to promoters, and negatively affect the expression of several PHO genes with varying effects^18,35,36^. We have assessed the effect of the RpoS and SpoT activity on the system by applying sensitivity analysis on the transcription rate of PhoB, as depicted in Fig. S3 for r14. In the light of this analysis, we have investigated the effect of stress response on the results depicted in Fig. 4, whereby the model is calibrated under varying stress response conditions due to *P*_*i*_ starvation. By repeating the analysis in Fig. 4 under these conditions, we have tested the activity of various promoter designs (Fig. S18). These results confirm the previous observations, and indicate that stress response can have a modulating effect, which can however be factored for by promoter design.

These results indicate that the steady state promoter activity and the PhoA yield are highly correlated in all the regimes and for all the promoter binding and unbinding rates. As expected, when the control system’s output in *P*_*i*_ starvation condition is compared with the output in regimes with increased external *P*_*i*_, we observe a decrease in PhoA yield. Moreover, these results predict that in order to obtain the starvation response in the conditions with higher external *P*_*i*_ concentration, promoter binding rates need to be increased and unbinding decreased. Promoters that provide the required strengths can be obtained by modifying the nucleotide sequences, for example, as in^28,37,38^.

**Figure 5:**
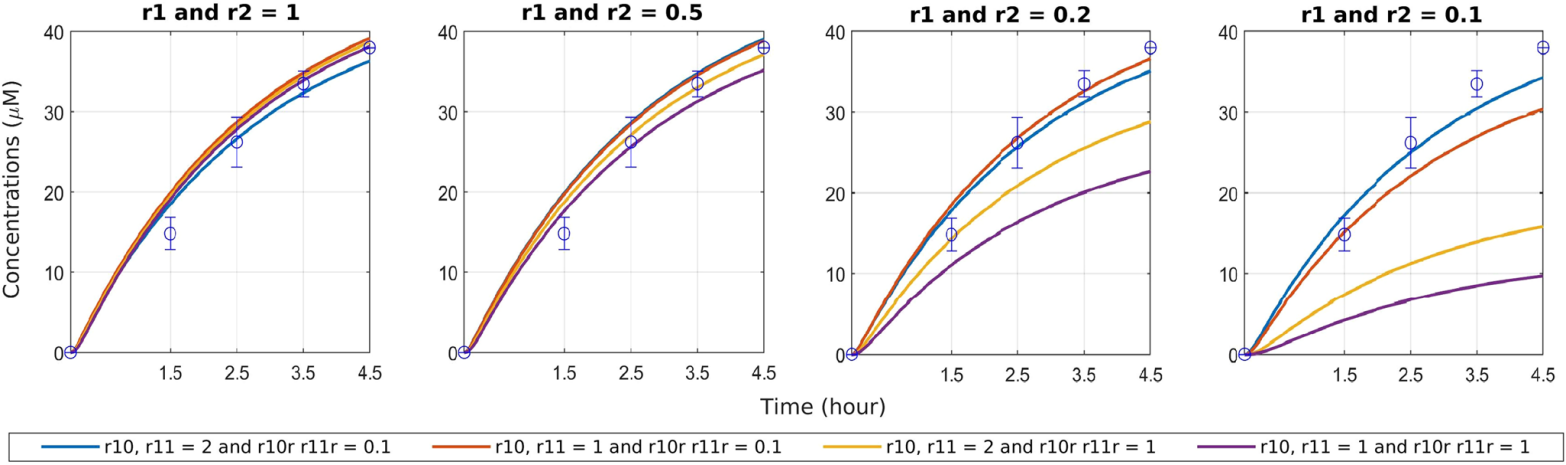
Comparison of the experimental data on PhoA expression with the synthetic promoter together with the simulation results with varying external *P*_*i*_ concentrations and promoter parameters that model synthetic designs. The experimental data on the starvation response with the synthetic promoter is represented as hollow circles. As in Fig. 4, the plots are ordered decreasingly from left to right according to the external *P*_*i*_ concentration given by the fold changes applied to the PhoR autphosphorylation reactions r1 and r2. The left most column with 1 as the fold change value is the starvation condition with 0 *μM* external *P*_*i*_. Each plot displays four simulations with varying fold change values applied to promoter binding and unbinding rates that model various promoter designs. A modified promoter (blue curve) can reproduce the starvation response in low as well as high external *P*_*i*_ concentration, and reproduce the experimental data under the synthetic promoter starvation conditions.

We have tested the predictions of the model on experimental data obtained with the synthetic promoter under starvation conditions with 0 *μM* external *P*_*i*_ concentration. Moreover, we have used the model to explore the effect of such synthetic promoters under various external *P*_*i*_ concentration conditions. The experimental data and the simulation results with our model are depicted in Fig. 5. The selected synthetic promoter verifies the model predictions as it has a similar behaviour as the control model for the pPhoA promoter in starvation conditions. Moreover, the model further predicts the synthetic promoters with increased strength, given with higher binding rates and lower unbinding rates, deliver responses similar to the starvation response, also in the presence of higher external *P*_*i*_ concentrations. Within a modular framework, the simulation results mechanistically quantify how changes in the genetic components affect the behaviour of the circuit.

#### Sensitivity Analysis

**Figure 6:**
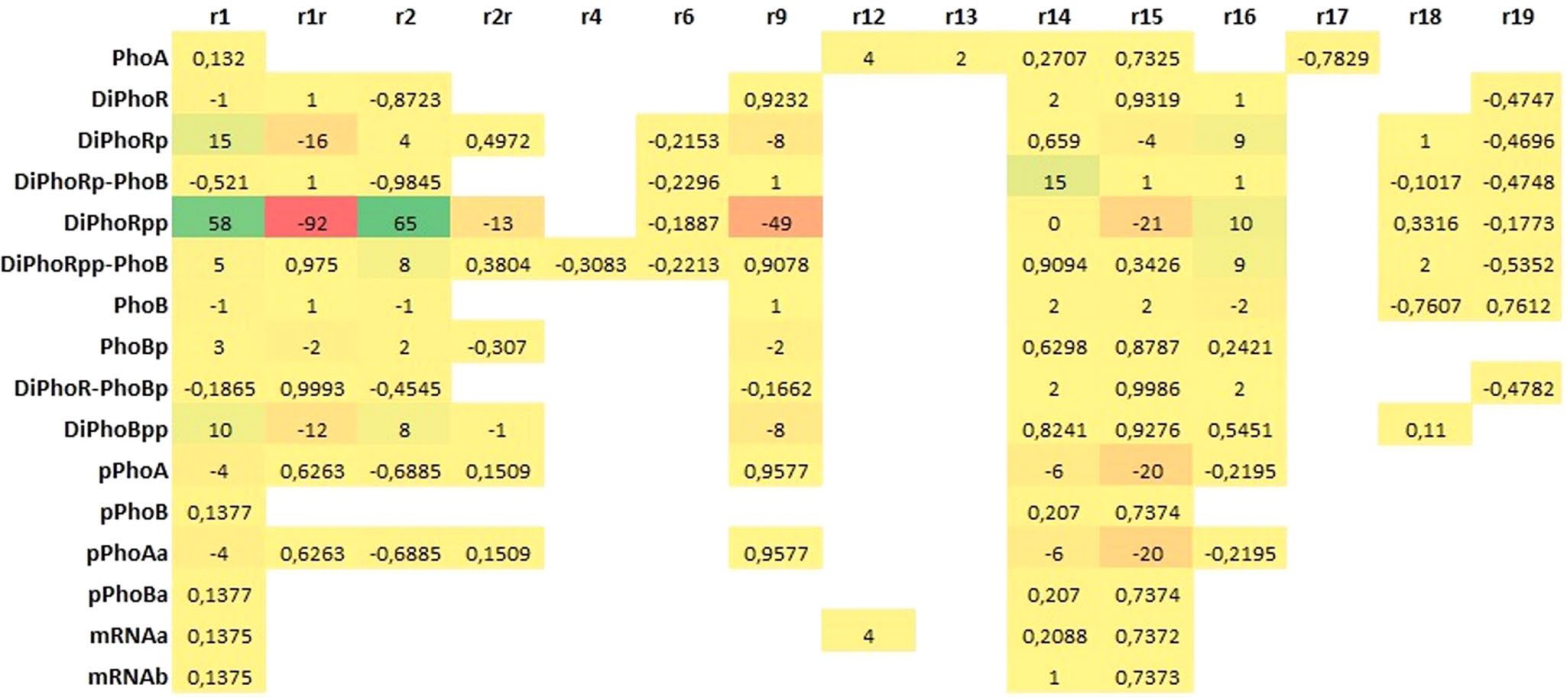
Heatmap displaying the results of the sensitivity analysis by considering the physiological interval in Table 1. For each parameter, the maximum and minimum values within its physiological range are considered for simulation, and the area under the curve (AUC) for each species is computed. The difference of the AUC for the maximum and minimum parameter values are then normalised with the AUC of the control model. Red represents the decreasing effect and green represents the increasing effect.

To assess the sensitivity of our model to the parameters, we have performed a two-step analysis. In the first step, we have considered the physiological interval of the parameters given in Table 1. For this analysis, we have only included the rate parameters that have been taken from literature and have been estimated within a given range. For each parameter, we have run simulations by instantiating the model with the maximum and minimum values of its physiological range, and, for each species, we have computed the yield of the system in terms of the area under the curve (AUC). Figure 6 displays the results obtained by taking the difference of the AUC for the maximum and minimum parameter values, normalised with the AUC of the control model. The resulting heatmap quantifies the impact of each parameter on the system dynamics with respect to plausible variations within its physiological range. The results demonstrate that the changes in the translation parameters are more pronounced than in the others. Moreover, in accordance with the results above, the autophosphorylation rate of PhoR impacts the active transcription factor levels and the transient species that lead to it.

In the second step, we have performed a sweeping analysis by considering all the model reactions and species within a range of 3 orders of magnitude both up and down. That is, each reaction rate is multiplied with a fold-change factor within a spectrum of 6 orders of magnitude, that is, from 10^−3^ to 10^3^. We have then computed the AUC for each species and normalised the outcome with the AUC of the control model. The heatmap depicted in Fig. S19 quantifies the impact of these changes and predicts the system behaviour under hypothetical conditions simulated by such variations in parameters.

## Discussion

We have presented a computational model and its experimental validation for quantifying dynamic mechanisms of auto-regulation in *Escherichia coli* in response to external *P*_*i*_ levels. Our model provides a mechanistic explanation of the interplay between transcriptional regulatory network, given by a two-component system (TCS) and Pho regulon, and promoter efficiency under variations in external *P*_*i*_ concentrations. Being parameterised within the physiological ranges of its components, the output of the model in terms of gene regulation delivers the expected system dynamics. The results provided by the model are in good agreement with the theory and the general concepts described in the literature for the functionality of Pho regulon^2,3^. The model provides predictions for the complex effects of TCS activity and consequent dynamics, including synthetic promoters with varying affinities to their transcription factor. A direct validation of the predictions is provided by the good fit of the experimental data.

With these insights, this work offers a mechanistic understanding of inorganic phosphate intake, realised by Pho regulon in a way that connects signalling with the genetic level. It provides a quantitative description of how different proteins interact to form a biological control system. It also describes the control of the phosphate-starvation response at the genetic level. Our work provides measurements of protein, phosphorylation, and promoter activity levels that are fundamental to define features of TCS circuits. One of the major outcomes is that our results do not only explain the observed experimental data, but also provide predictions on the physiology of the Pho regulon and insights for the synthetic promoter design. Moreover, our work has implications for applications in artificial life and for others in biotechnology that exploit such mechanisms. As the model architecture is intrinsically open to integrate supplementary layers, together with experimental findings, it should provide insights in investigations on engineering new dynamic sensors and regulators for living technologies.

Our model describes the responsive structure of the TCS system, which can be used to synthesize bacteria that detect changes in their environment and respond by modulating the synthesis and intake. Applications of such an engineering approach for bacteria should provide a basis for a new generation of bio-materials. We have presented a recipe for the design process of such an application, which integrates gene expression data from *E*. *coli* into a computer model.

Our model, which includes 29 reactions that describe the dynamic behaviour of the key regulatory network components, provides a quantification of the phosphate starvation response by means of both deterministic and stochastic simulations. The deterministic simulations allowed us to estimate the missing parameters of the model and analyse the mean behaviour of the system dynamics. In particular, we have exposed the phosphorylation cycle in TCS signalling and its role in the positive feedback mechanism in determining the network yield in terms of PhoA. Moreover, the steady state properties of the system could be displayed by sensitivity analysis on deterministic simulations.

In accordance with the common practice, we have employed stochastic simulations to highlight the effect of noise in the system^39^. As expected, the stochastic simulations are consistent with the deterministic simulations, which display the mean behaviour. Stochastic simulations, on the other hand, capture the noise due to small species numbers and concomitant fast and slow reactions. As a result of this, stochastic simulations with our model display the fluctuations observed in experimental observations, thereby exposing the fluctuations due to binding and unbinding of the transcription factors with the promoters, which operate at a much faster time scale in comparison to the preceding signalling cascade^40–42^. In these simulations, higher unbinding rates result in greater fluctuations that correlate with the decrease in binding saturation, a requirement for a robust signal. While confirming the notion that the complex networks of interacting molecules within cells should be robust^43,44^, these results highlight the additional role of the transcription factor unbinding rate in tuning the protein synthesis. Our stochastic simulation results, together with the experimental data on PhoB concentration in wild-type, suggest that the noise at the level of promoters controls the phenotypic variability in the mean behaviour. Large levels of noise at the level of PhoA and PhoB promoters are results of DNA unbinding rates. Moreover, our model shows that the smaller DNA unbinding rates also reduce the response time.

Our results confirm that the dynamics of the TCS and its responsiveness to both genetic and environmental perturbations play a key role in tuning the *E*. *coli P*_*i*_ response. In this respect, with parameter values obtained by fitting the experimental data on PhoA and PhoB promoters, we have investigated and identified the response dynamics of individual system species. Moreover, we have analysed synthetic promoter affinities that reproduce the wild type response to *P*_*i*_ starvation. For this purpose, we have tested and verified the model with experimental data for the response regulator binding affinity to infer the functional relation between the fraction of bound response regulator and the transcriptional activity. We have then quantified the effect of each protein in the pathway, through sensitivity analysis, singling out the main regulator mechanisms of the TCS.

Our results indicate the extent to which synthetic promoters can be tuned for various TCS detection thresholds such that the organism adapts to various environmental conditions. In this regard, because our model includes mechanisms for quantifying the information *E*. *coli* has about its phosphate environment, the model can be used to perform further experiments on the *P*_*i*_ intake system. Moreover, it can be used to explore and test various promoter designs, for example, in biotechnology applications such as sensors for wastewater treatment or detecting environmental pollutants to relocate towards them.

In conclusion, our study reveals how the tight interplay between theory and wet-lab experiments greatly helps to improve our understanding of bacterial sensing and signalling pathways. In particular, the TCS component of our model, which relays the signal on environmental changes to the genetic components for tuning protein expression, belongs to one of the largest and most diverse families of sensory components in biology. In this respect, the TCS features examined in this work provide a template for the models of similar systems that regulate the response for various external signals. An improved and quantitative understanding of such systems by formal mechanistic models will likely contribute to our understanding of the engineering of biosensors for diverse synthetic biology applications.

## Methods

The model is based on a mechanistic description of the system dynamics within a chemical reaction network representation with respect to mass action kinetics. To build the simulation model, we have selected the part of the system from the TCS to gene regulation, and used the promoter activity as well as the levels of active transcription factor as indicators for quantifying the response to external *P*_*i*_ levels.

In the following, to better illustrate the response of Pho regulon and TCS signalling to the *P*_*i*_ starvation, we describe the network in terms of the interactions of system components as a chemical reaction network. Figure 2B provides an alternative representation of the regulatory system interactions given by the chemical reaction network.

When the external *P*_*i*_ concentration is limited, PhoR is free to bind ATP. This allows PhoR to autophosphorylate itself. PhoR is stable as a dimer, which is denoted by DiPhoR. Therefore, it is doubly phosphorylated.

1 $${\rm{DiPhoR}}\leftrightarrow {\rm{DiPhoRp}}$$

2 $${\rm{DiPhoRp}}\leftrightarrow {\rm{DiPhoRpp}}$$

PhoR is essential for the control of the activity of PhoB^2,8,45^. It phosphorylates PhoB through an autokinase/phosphotransferase activity^2,8^. After autophosphorylating, PhoR relays the signal to the transcription factor PhoB. The bidirectional reactions 3 and 5 below model the association of phosphorylated PhoR dimer and PhoB, and the unidirectional reactions 4 and 6 model the phosphotransferase. PhoB has been reported to exist primarily as monomers and phosphorylation greatly enhances dimerisation of PhoB (DiPhoBpp), modelled by reaction 7^10^.

3 $${\rm{DiPhoRpp}}+{\rm{PhoB}}\leftrightarrow {\rm{DiPhoRpp}}\,-\,{\rm{PhoB}}$$

4 $$\mathrm{DiPhoRpp}-\mathrm{PhoB}\to {\rm{DiPhoRp}}+{\rm{PhoBp}}$$

5 $${\rm{DiPhoRp}}+{\rm{PhoB}}\leftrightarrow {\rm{DiPhoRp}}\,-\,{\rm{PhoB}}$$

6 $${\rm{DiPhoRp}}\,-\,{\rm{PhoB}}\to {\rm{DiPhoR}}+{\rm{PhoBp}}$$

7 $${\rm{PhoBp}}+{\rm{PhoBp}}\leftrightarrow {\rm{DiPhoBpp}}$$

In *E*. *coli*, the sensor histidine kinase PhoR is a bifunctional enzyme that paradoxically performs two opposed tasks: in one direction, it catalyzes the phosphorylation of response regulator PhoB, and in the other, it also performs the dephosphorylation of phosphorylated PhoB, which is PhoBp^18,25^. The association of PhoR dimers with phosphorylated PhoB is modelled by the bidirectional reaction 8, whereas the phosphatase activity is given by reaction 9.

8 $${\rm{DiPhoR}}+{\rm{PhoBp}}\leftrightarrow {\rm{DiPhoR}}\,-\,{\rm{PhoBp}}$$

9 $${\rm{DiPhoR}}\,-\,{\rm{PhoBp}}\to {\rm{DiPhoR}}+{\rm{PhoB}}$$

Phosphorylated dimer structure PhoB (DiPhoBpp) is enabled for activating Pho regulon by binding to a consensus promoter region. PhoB and PhoR in Pho regulon are encoded by the same operon, that is, the phoBR operon. Thus, the synthesis of the regulatory proteins PhoB and PhoR is under Pho regulon control^2,8,46^.

Based on experimental data, we consider the PhoA and PhoB promoters (pPhoA, pPhoB), whereby the PhoB promoter provides feedback to the system as this results in the expression of both PhoB and PhoR. PhoR expression during *P*_*i*_ limitation is dependent on the upstream pPhoB; the operon structure indicates that PhoR gene function requires expression from the pPhoB^46^. For this, phosphorylated PhoB dimers (DiPhoBpp) bind to the promoter as active transcription factors.

10 $${\rm{DiPhoBpp}}+{\rm{pPhoA}}\leftrightarrow {\rm{pPhoAa}}$$

11 $${\rm{DiPhoBpp}}+{\rm{pPhoB}}\leftrightarrow {\rm{pPhoBa}}$$

Active promoters pPhoAa and pPhoBa lead to the transcription of mRNA, which carry the information for the subsequent translation, resulting in protein synthesis, which are PhoA, PhoB and DiPhoR^2,8^.

12 $${\rm{pPhoAa}}\to {\rm{pPhoAa}}+{\rm{mRNAa}}$$

13 $${\rm{mRNAa}}\to {\rm{PhoA}}+{\rm{mRNAa}}$$

14 $${\rm{pPhoBa}}\to {\rm{pPhoBa}}+{\rm{mRNAb}}$$

15 $${\rm{mRNAb}}\to {\rm{PhoB}}+{\rm{mRNAb}}$$

16 $${\rm{mRNAb}}\to {\rm{DiPhoR}}+{\rm{mRNAb}}$$

With the inclusion of the degradation/dilution terms, we obtain:

17 $${\rm{PhoA}}\to \varnothing $$

18 $${\rm{PhoB}}\to \varnothing $$

19 $${\rm{DiPhoR}}\to \varnothing $$

20 $${\rm{mRNAa}}\to \varnothing $$

21 $${\rm{mRNAb}}\to \varnothing $$

We have implemented the deterministic ordinary differential equation (ODE) model in Matlab by using the standard translation from the chemical reaction network above, based on stoichiometries. The ODEs and detailed descriptions of each system variable are listed in Fig. S1 and Table S1. We have also implemented a version of the model for stochastic simulation, again based on mass action kinetics. The stochastic simulations capture fluctuations due to small molecule numbers, which are not captured by the deterministic simulations. By using the standard conversion factors for mass action kinetics, we could use the same rates for the deterministic and stochastic simulations.

The initial concentrations of the model variables have been derived from the literature or obtained from our experiments, described below. The control model has been calibrated for an initial culture containing 0 *μM* external *P*_*i*_. Prior to *P*_*i*_ starvation, the concentrations of proteins PhoR and PhoB are approximately 0.22 *μM*. The concentrations of active PhoR and active PhoB are $4\cdot {10}^{-8}\,\mu M$ and $6\cdot {10}^{-8}\,\mu M$, as determined by Keasling *et al*.^3^. With a single plasmid, average mRNA number is 2–3 in *E*. *coli*^47^. Therefore, the initial states of mRNAa and mRNAb are set to 0.00166 *μM* by taking *E*. *coli* volume as 1 *μm*^3^, and the initial promoters numbers are set to 10 for each. We assume that the ATP concentration stays constant throughout the considered time intervals.

The rates of chemical reactions are obtained in accordance with the variability of physiological ranges given in the literature^5,6,47–50^. The model includes 29 reactions, including the reverse reactions. The control model has been used to reproduce the data and the unknown parameters have been estimated by least square inference within the plausible physiological ranges. When possible, parameter values are fixed or estimated by using experimental measurements found in the literature. The parameter values taken from the literature and their physiological ranges for the rates, if applicable, are listed in Table 1. The parameters without a range are fitted to the experimental data by using the deterministic model to reproduce the response curves. The parameter estimation procedure has been carried out by using a multi-start approach. The rate values have been selected with respect to the best fit to the physiological ranges, also listed in Table 1, and the dynamics in accordance with the experimental findings in order to avoid discontinuities or states with unrealistic values.

The data for the PhoA and PhoB expression have been obtained using PCR amplified DNA from *E*. *coli* MG1655 genome and transcriptionally fused to the translational coupler BCD2^51^ and the fluorescent *ms-fgfp* gene. Subsequently the PphoA-BCD2-msfgpf and PphoB-BCD2-msfgfp fragments were cloned using the PacI/HindIII restrictions sites in pSEVA234 plasmid (http://seva.cnb.csic.es/), generating the pSEVA237PphoA and pSEVA237PphoB vectors as depicted in Fig. 2A.

The synthetic promoter Pliar was obtained as in^52,53^ with modifications. Activities of the PphoA and PphoB promoters have been determined as follows. Cells were maintained on LB rich medium. For Pi assays, an overnight preculture of *E*. *coli* DH10B carrying pSEVA237PphoA, pSEVA237PphoB or pSEVA237PLiar00117 plasmids harbouring PhoA, PhoB or PLiar00117 promoters driving the expression of MsfGFP, respectively were grown in 10 *ml* MOPS medium (pH = 7.2), supplemented with 0.02% casamino acids and 5 *mM* KH_2_PO_4_ at 37 °C under constant shaking at 250 rpm. The activity of PhoB and PhoA promoters were analysed in resting cells, both in presence or absence of 50 *μg*/*ml* antibiotic kanamycin, which did not have any impact on the promoter activity as it can be seen in the Supplementary Figs S2 and S20. The bacterial precultures were used to inoculate 50 *ml* of the same medium reaching an OD600 of 0.1 and grown until mild-late exponential phase (OD600 of 0.6–0.9). At this point, the bacterial cells were pelleted at 1500 × g for 10 min at room temperature, and finally washed twice in MOPS medium without ${{{\rm{PO}}}_{4}}^{\underline{3-}}$. Subsequently, the cells were suspended in 250 *μl* of MOPS to reach a final OD600 of 2 with increasing concentrations of ${{{\rm{PO}}}_{4}}^{\underline{3-}}$ (from 0 to 50 *mM*). The bacterial cell suspensions were loaded in 96-well plates and the expression of MsfGFP (fluorescence) was measured at different times in a SpectraMax i3x (Molecular Device) at 30 °C. The excitation wavelength was set to 485 *nm* and the fluorescence emission was measured at different times at 525 *nm*.

## Supplementary information

Supplementary file

Matlab model file
