# Supplementary material for: Quantifying dynamic mechanisms of auto-regulation in Escherichia coli with synthetic promoter in response to varying external phosphate levels
Source: Sci Rep. 2019 Feb 14;9:2076. doi: 10.1038/s41598-018-38223-w (PMC6376016; doi:10.1038/s41598-018-38223-w)
Supplement: Supplementary file 1 — Supplementary file [file 41598_2018_38223_MOESM1_ESM.pdf]

Quantifying dynamic mechanisms of auto-regulation in Escherichia coli with synthetic promoter in response to varying external phosphate levels

– Supplementary Information –

Cansu Uluşeker[1,2], Jesús Torres-Bacete[3], José L. García[4,5], Martin M. Hanczyc[1,6], Juan Nogales[3], Ozan Kahramanoğulları[7]

[1] Centre for Integrative Biology, University of Trento, Trento, Italy

[2] The Microsoft Research – University of Trento Centre for Computational and Systems Biology, Rovereto, Italy

[3] Systems Biology Department, Centro Nacional de Biotecnología (CNB-CSIC), 28049 Madrid, Spain

[4] Microbial and Plant Biotechnology Department, Centro de Investigaciones Biolgicas (CIB-CSIC), 28040 Madrid, Spain

[5] Applied Systems Biology and Synthetic Biology Department, Institute for Integrative Systems Biology (I^{2}Sysbio-CSIC-UV), 46980 Paterna, Spain

[6] Chemical and Biological Engineering, University of New Mexico, USA

[7] Department of Mathematics, University of Trento, Trento, Italy

ozan.kahramanogullari@unitn.it

Variables	Description
PhoA	Alkaline phosphatase PhoA
DiPhoR	Dimer structure of sensor histidine kinase PhoR
DiPhoRp	Phosphorylated dimer structure of PhoR
DiPhoRpp	Doubly phosphorylated dimer structure of PhoR
PhoB	Monomer structure of response regulator PhoB
DiPhoRpp-PhoB	Bound form of DiPhoRpp and PhoB for phosphotransferase
PhoBp	Phosphorylated monomer structure of response regulator PhoB
DiPhoRp-PhoB	Bound form of DiPhoRp and PhoB for phosphotransferase
DiPhoBpp	Doubly phosphorylated dimer structure of PhoB
DiPhoR-PhoBp	Bound form of DiPhoR and PhoBp for phosphatase
pPhoA	PhoA promoter
pPhoAa	Active PhoA promoter
pPhoB	PhoB promoter
pPhoBa	Active PhoB promoter
mRNAa	mRNA of PhoA
mRNAb	mRNA of PhoB

Table 1: List of variables in the model

$$\mathrm{d}Gfp(t)/\mathrm{d}t = r_{18}.mRNAa(t) - r_{22}.Gfp(t)$$

$$\begin{aligned}\mathrm{d}DiPhoR(t)/\mathrm{d}t = {} & r_{6r}.DiPhoRp(t) - r_{6}.DiphoR(t)\\ & +r_{11}.DiPhoRp\text{-}PhoB(t) + r_{21}.mRNAb(t)\\ & -r_{24}.DiPhoR(t) - r_{13}.DiPhoR(t).PhoBp(t)\\ & +r_{13r}.DiPhoR\text{-}PhoBp(t) + r_{14}.DiPhoR\text{-}PhoBp(t)\end{aligned}$$

$$\begin{aligned}\mathrm{d}DiPhoRp(t)/\mathrm{d}t = {} & r_{6}.DiPhoR(t) - r_{6r}.DiPhoRp(t)\\ & -r_{7}.DiPhoRp(t) + r_{7r}.DiPhoRpp(t)\\ & +r_{9}.DiPhoRpp\text{-}PhoB(t) + r_{10r}.DiPhoRp\text{-}PhoB(t)\\ & -r_{10}.DiPhoRp(t).PhoB(t)\end{aligned}$$

$$\begin{aligned}\mathrm{d}DiPhoRp\text{-}PhoB(t)/\mathrm{d}t = {} & r_{10}.DiPhoRp(t).PhoB(t)\\ & -r_{10r}.DiPhoRp\text{-}PhoB(t) - r_{11}.DiPhoRp\text{-}PhoB(t)\end{aligned}$$

$$\begin{aligned}\mathrm{d}DiPhoRpp(t)/\mathrm{d}t = {} & r_{7}.DiPhoRp(t) - r_{7r}.DiPhoRpp(t)\\ & -r_{8}.DiPhoRpp(t).PhoB(t) + r_{8r}.DiPhoRpp\text{-}PhoB(t)\end{aligned}$$

$$\begin{aligned}\mathrm{d}DiPhoRpp\text{-}PhoB(t)/\mathrm{d}t = {} & r_{8}.DiPhoRpp(t).PhoB(t)\\ & -r_{8r}.DiPhoRpp\text{-}PhoB(t) - r_{9}.DiPhoRpp\text{-}PhoB(t)\end{aligned}$$

$$\begin{aligned}\mathrm{d}PhoB(t)/\mathrm{d}t = {} & -r_{8}.DiPhoRpp(t).PhoB(t)\\ & +r_{8r}.DiPhoRpp\text{-}PhoB(t) - r_{10}.DiPhoRp(t).PhoB(t)\\ & +r_{10r}.DiPhoRp\text{-}PhoB(t) + r_{20}.mRNAb(t)\\ & -r_{23}.PhoB(t) + r_{14}.DiPhoR\text{-}PhoBp(t)\end{aligned}$$

$$\begin{aligned}\mathrm{d}PhoBp(t)/\mathrm{d}t = {} & r_{9}.DiPhoRpp\text{-}PhoB(t) - 2.r_{12}.PhoBp(t)^{2}\\ & +r_{11}.DiPhoRp\text{-}PhoB(t) + 2.r_{12r}.DiPhoBpp(t)\\ & +r_{13r}.DiPhoR\text{-}PhoBp(t) - r_{13}.DiPhoR(t).PhoBp(t)\end{aligned}$$

$$\begin{aligned}\mathrm{d}DiPhoR\text{-}PhoBp(t)/\mathrm{d}t = {} & r_{13}.DiPhoR(t).PhoBp(t)\\ & -r_{13r}.DiPhoR\text{-}PhoBp(t) - r_{14}.DiPhoR\text{-}PhoBp(t)\end{aligned}$$

$$\begin{aligned}\mathrm{d}DiPhoBpp(t)/\mathrm{d}t = {} & r_{12}.PhoBp(t)^{2} - r_{12r}.DiPhoBpp(t)\\ & -r_{15}.DiPhoBpp(t).pPhoA(t) + r_{16r}.pPhoBa(t)\\ & +r_{15r}.pPhoAa(t) - r_{16}.DiPhoBpp(t).pPhoB(t)\end{aligned}$$

$$\mathrm{d}pPhoA(t)/\mathrm{d}t = -r_{15}.DiPhoBpp(t).pPhoA(t) + r_{15r}.pPhoAa(t)$$

$$\mathrm{d}pPhoAa(t)/\mathrm{d}t = r_{15}.DiPhoBpp(t).pPhoA(t) - r_{15r}.pPhoAa(t)$$

$$\mathrm{d}pPhoB(t)/\mathrm{d}t = -r_{16}.DiPhoBpp(t).pPhoB(t) + r_{16r}.pPhoBa(t)$$

$$\mathrm{d}pPhoBa(t)/\mathrm{d}t = r_{16}.DiPhoBpp(t).pPhoB(t) - r_{16r}.pPhoBa(t)$$

$$\mathrm{d}mRNAa(t)dt = r_{17}.pPhoAa(t) - r_{25}.mRNAa(t)$$

$$\mathrm{d}mRNAb(t)/\mathrm{d}t = r_{19}.pPhoBa(t) - r_{26}.mRNAb(t)$$

Figure 1: The ODEs for the model reactions.

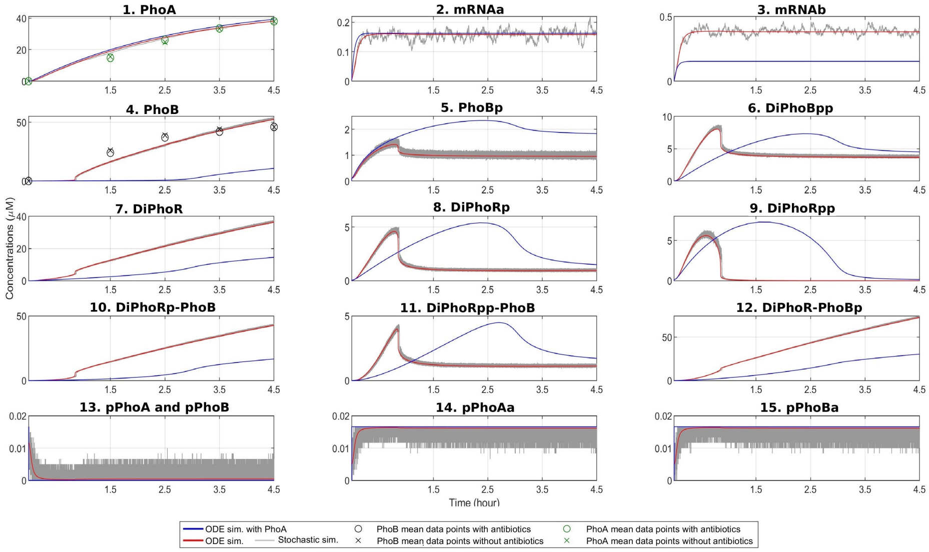

Figure 2: The deterministic ODE simulation results that are fitted with only the experimental data on PhoA expression levels are plotted in blue. The deterministic ODE simulation results that are fitted with the experimental data on both PhoA and PhoB expression levels are plotted in red. In all the experiments, the external P_i concentration is $0\mu M$ and a time-course of 4.5 hours is considered. We have analyzed the PhoB and PhoA promoters activity by following the expression of MsfGFP by fluorescence. Their activity has been analyzed in resting cells, both in the presence or absence of $50\mu g/ml$ kanamycin (Km), whereby Km did not have any impact on the promoter activity: as it can be seen in the supplementary Figure S20, there is no difference in the PhoA and PhoB activity, irrespective of the presence or absence of Km. For the simulations that consider both PhoA and PhoB data, stochastic simulation results, plotted in grey, are also provided. In the simulations that include both of the PhoA and PhoB data, the values for r14 and r15 in Table 1 in the main text are obtained by increasing these parameters from 0.05 to 0.13 and from 0.03 to 0.035, respectively. Similarly, the disassociation rates, r3r, r7r, and and r8r are decreased from 74.9411 to 44.9411, 24.9411 and 34.9411 and r5r is increased from 74.94 to 94.94 to obtain comparable DiPhoBpp levels.

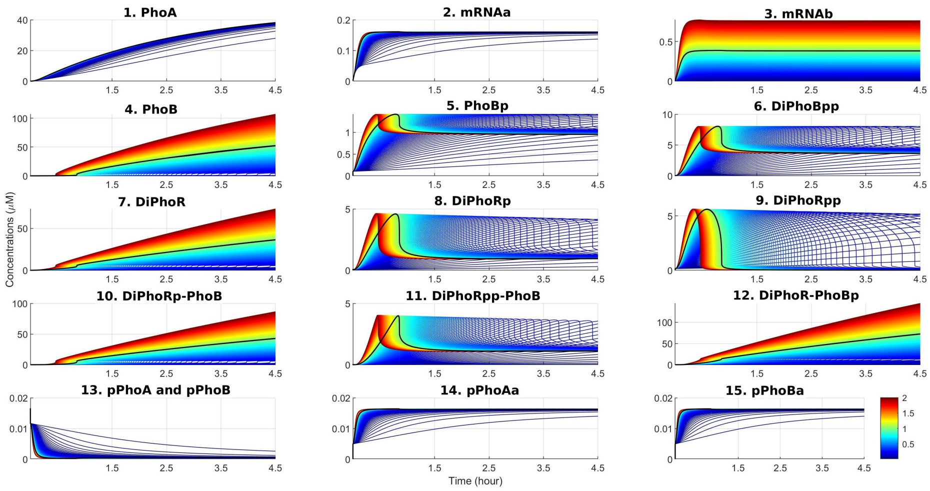

Figure 3: The cumulative output of simulations under the effect of random perturbations on the reaction rate r14. The reaction rates in the model can vary within a range in different cell types and cellular environments. Therefore, we applied sensitivity analysis to quantify the model outputs with respect to such random perturbations. The rate is modified by applying a varied range of fold changes between 0.01 to 2, which are larger than the physiological range. The 1 fold change is plotted in black.

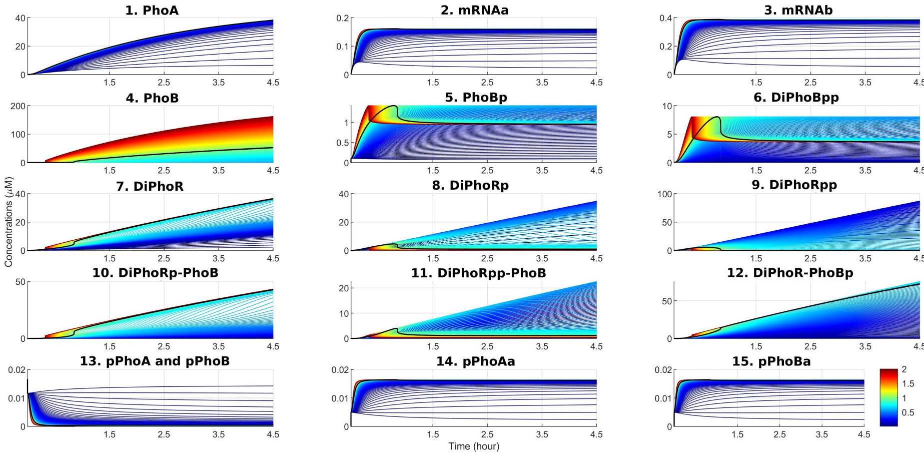

Figure 4: The cumulative output of simulations under the effect of random perturbations on the reaction rate r15. The reaction rates in the model can vary within a range in different cell types and cellular environments. Therefore, we applied sensitivity analysis to quantify the model outputs with respect to such random perturbations. The rate is modified by applying a varied range of fold changes between 0.01 to 2, which are larger than the physiological range. The 1 fold change is plotted in black.

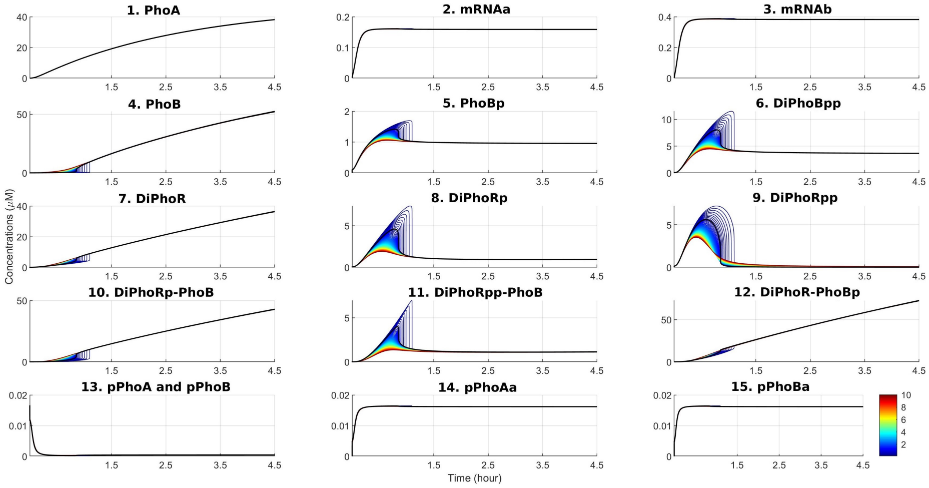

Figure 5: The cumulative output of simulations under the effect of random perturbations on the reaction rate r3r. The reaction rates in the model can vary within a range in different cell types and cellular environments. We applied sensitivity analysis to quantify the model outputs with respect to such random perturbations. The rate is modified by applying a varied range of fold changes between 0.1 to 10. The 1 fold change is plotted in black.

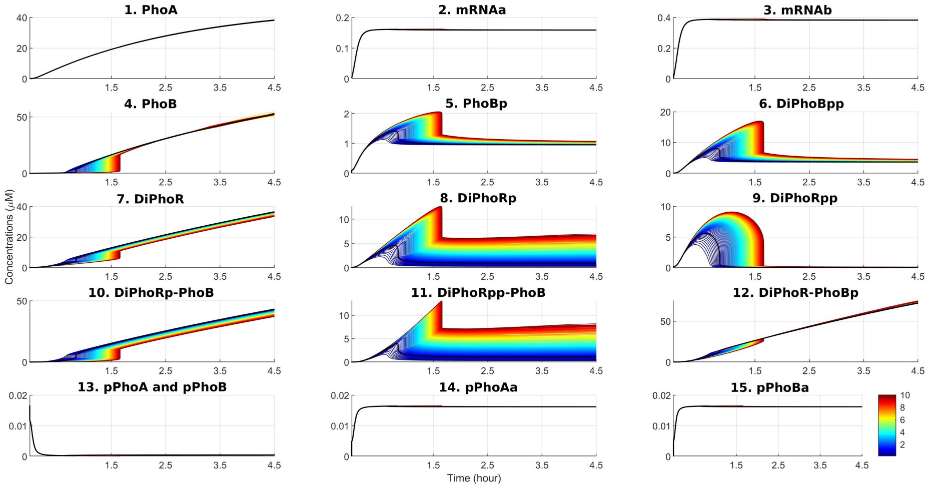

Figure 6: The cumulative output of simulations under the effect of random perturbations on the reaction rate r5r. The reaction rates in the model can vary within a range in different cell types and cellular environments. We applied sensitivity analysis to quantify the model outputs with respect to such random perturbations. The rate is modified by applying a varied range of fold changes between 0.1 to 10. The 1 fold change is plotted in black.

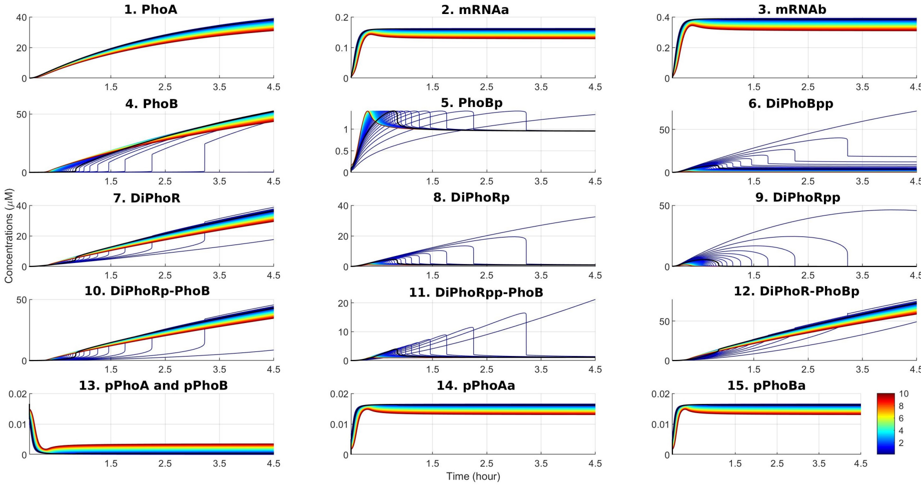

Figure 7: The cumulative output of simulations under the effect of random perturbations on the reaction rate r7r. The reaction rates in the model can vary within a range in different cell types and cellular environments. We applied sensitivity analysis to quantify the model outputs with respect to such random perturbations. The rate is modified by applying a varied range of fold changes between 0.1 to 10. The 1 fold change is plotted in black.

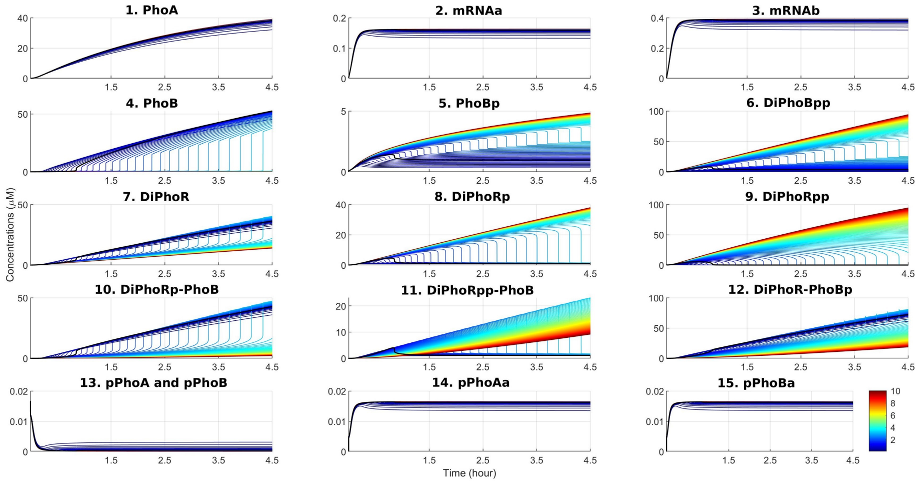

Figure 8: The cumulative output of simulations under the effect of random perturbations on the reaction rate r8r. The reaction rates in the model can vary within a range in different cell types and cellular environments. We applied sensitivity analysis to quantify the model outputs with respect to such random perturbations. The rate is modified by applying a varied range of fold changes between 0.1 to 10. The 1 fold change is plotted in black.

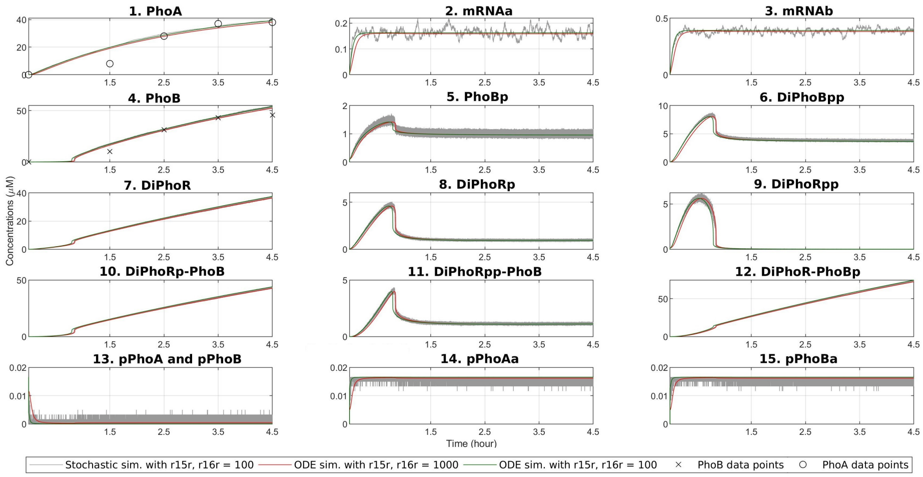

Figure 9: Stochastic simulation results when DNA unbinding rates r10 and r11 are set to $100\,s^{-1}$ together with deterministic simulation results. The unbinding kinetics can have a significant impact on the stochastic dynamics of gene expression network. When the promoter unbinding rate increases, the promoter activity, which works at a faster time-scale, leads to much more noisy fluctuations (see Figures S10 and S11). A stochastic simulation with this modified rate is plotted in gray. The deterministic simulations with our model (red) and the deterministic simulations with the changes above (green) display similar dynamics.

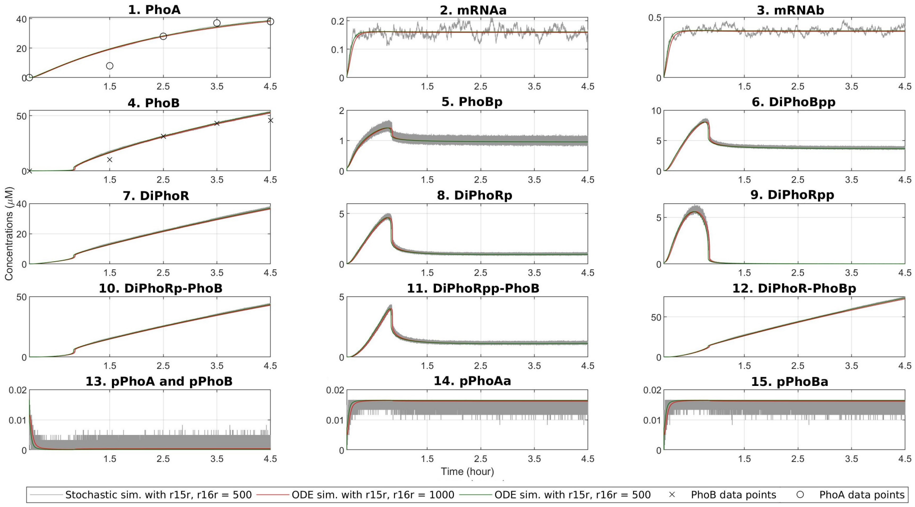

Figure 10: Stochastic simulation results when DNA unbinding rates r10 and r11 are set to $500\,s^{-1}$ together with deterministic simulation results. The unbinding kinetics can have a significant impact on the stochastic dynamics of gene expression network. When the promoter unbinding rate increases, the promoter activity, which works at a faster time-scale, leads to much more noisy fluctuations (see Figures S9 and S11). A stochastic simulation with this modified rate is plotted in gray. The deterministic simulations with our model (red) and the deterministic simulations with the changes above (green) display similar dynamics.

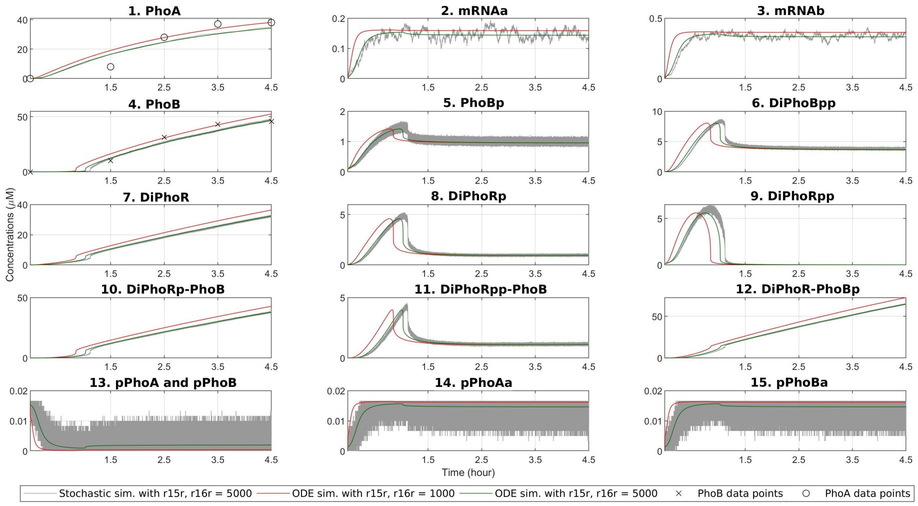

Figure 11: Stochastic simulation results when DNA unbinding rates r10 and r11 are set to $5000\,s^{-1}$ together with deterministic simulation results. The unbinding kinetics can have a significant impact on the stochastic dynamics of gene expression network. When the promoter unbinding rate increases, the promoter activity, which works at a faster time-scale, leads to much more noisy fluctuations (see Figures S9 and S10). A stochastic simulation with this modified rate is plotted in gray. The deterministic simulations with the changes above with slower unbinding rates (green) lead to slightly slower dynamics in comparison to deterministic simulations with our model with this rate set to $1000\,s^{-1}$ (red).

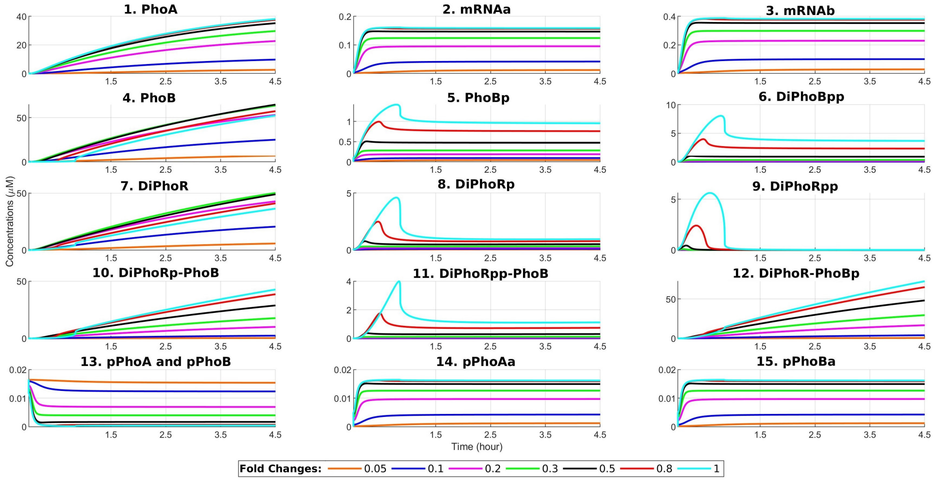

Figure 12: Simulation results with varying external P_i concentrations. A decrease in the external P_i concentration is modelled as a fold change, listed in the legend, which is applied to the autophosphorylation reactions of PhoR. A fold change of 1 gives the starvation condition with $0\mu M$ external P_i. A lower fold change value represents an increased PhoU activity. Active PhoU inhibits PhoR, and thereby prevents it from autophosphorylating, which otherwise relays the signal downstream to the promoters pPhoA and pPhoB.

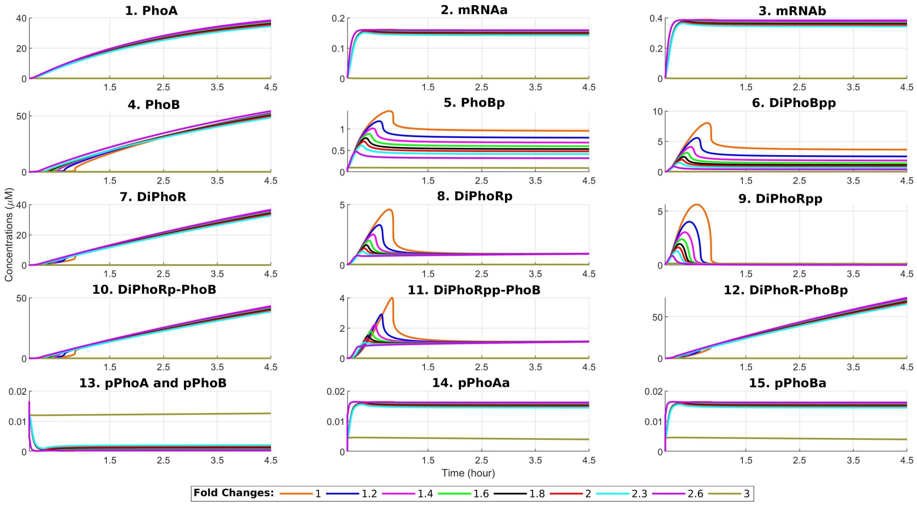

Figure 13: Deterministic simulations with the model with varying fold changes applied to r8, the association rate of PhoR and PhoB, whereby PhoR acts as a phosphatase. The plots display the effect of the perturbations on DiPhoBpp in comparison to the case, where the fold change is 1.

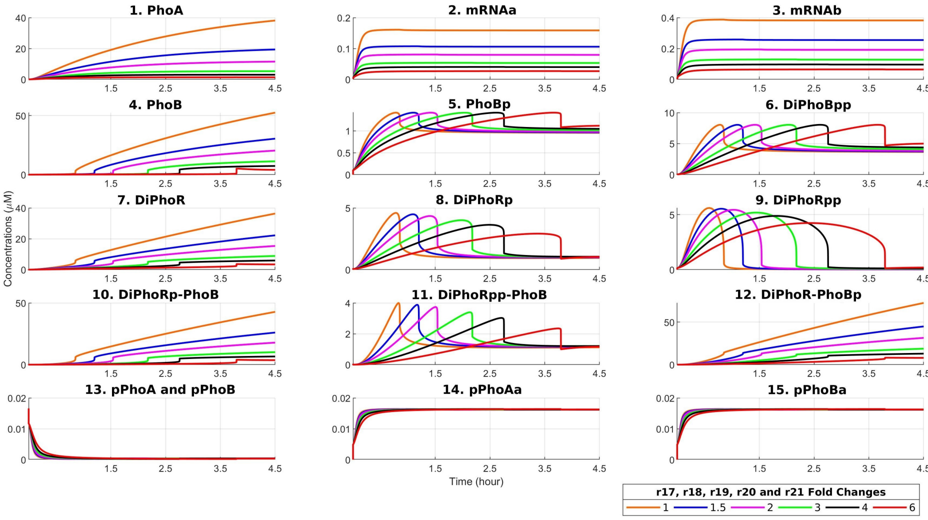

Figure 14: Deterministic simulation with the model with varying fold changes applied to degradation and dilution terms r17, r18, r19, r20, and r21. Together with the Figures S15 and S16, the plots display the effect of degradation and dilution under the conditions of reduced phosphorylation rates for PhoR resulting from an increase in external P_i concentration.

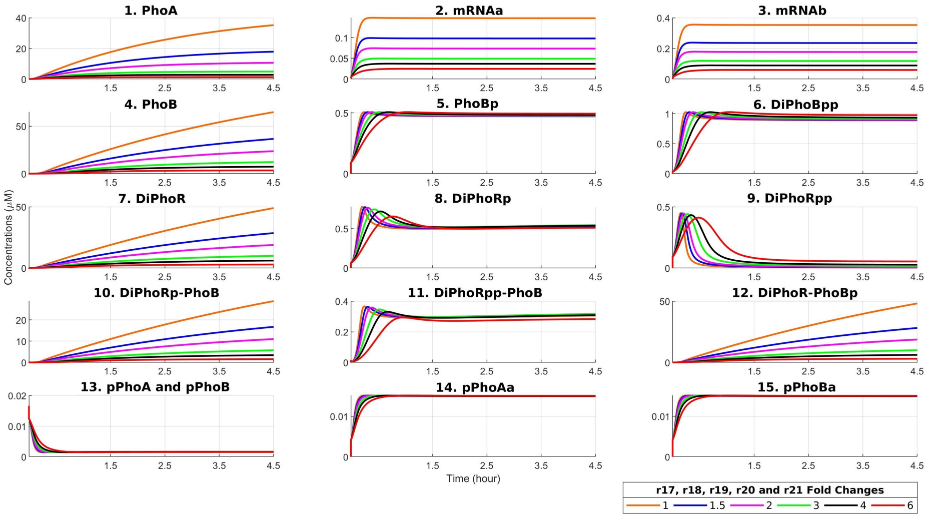

Figure 15: Deterministic simulation with the model with varying fold changes applied to degradation and dilution terms r17, r18, r19, r20, and r21 with a concomitant fold change of 0.5 applied to r1 and r2. Together with the Figures S14 and S16, the plots display the effect of degradation and dilution under the conditions of reduced phosphorylation rates for PhoR resulting from an increase in external P_i concentration.

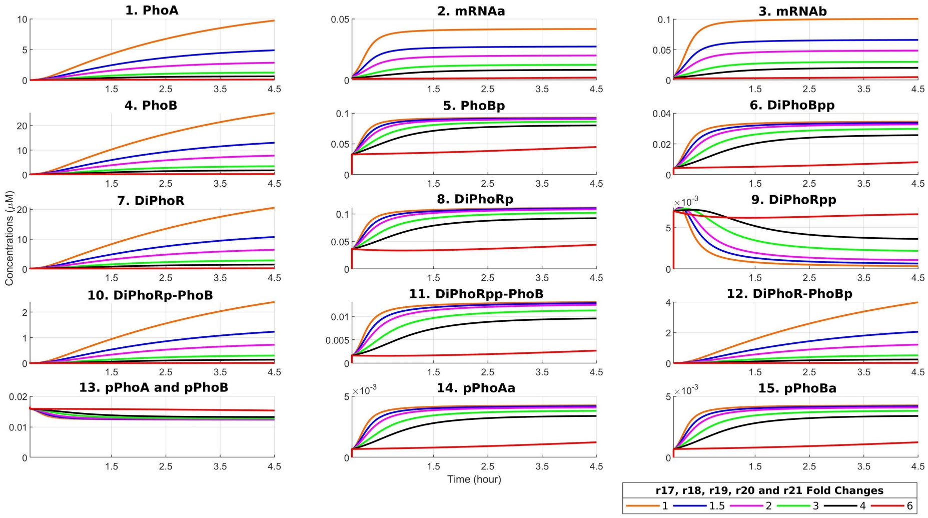

Figure 16: Deterministic simulation with the model with varying fold changes applied to degradation and dilution terms r17, r18, r19, r20, and r21 with a concomitant fold change of 0.1 applied to r1 and r2. Together with the Figures S14 and S15, the plots display the effect of degradation and dilution under the conditions of reduced phosphorylation rates for PhoR resulting from an increase in external P_i concentration.

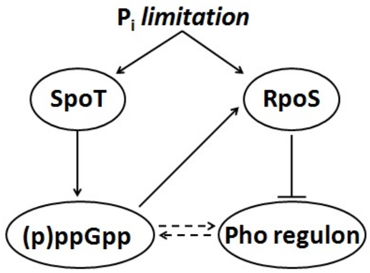

Figure 17: Interactions between the Pho regulon, RpoS sigma factor and stringent response in Escherichia coli. The arrows represent activation/expression (black head) and inhibition (T head). Dashed lines represent interactions that can be either direct or indirect. When external P_i is limited, stress responses are activated. P_i limitation induces accumulations of SpoT-dependent ppGpp and the rpoS-dependent RpoS protein, the sigma factor. Raised levels of sigma factor decrease the expression of Pho regulon. Under starvation conditions, SpoT is involved in elevating ppGpp levels, which in turn has a positive role in RpoS expression.

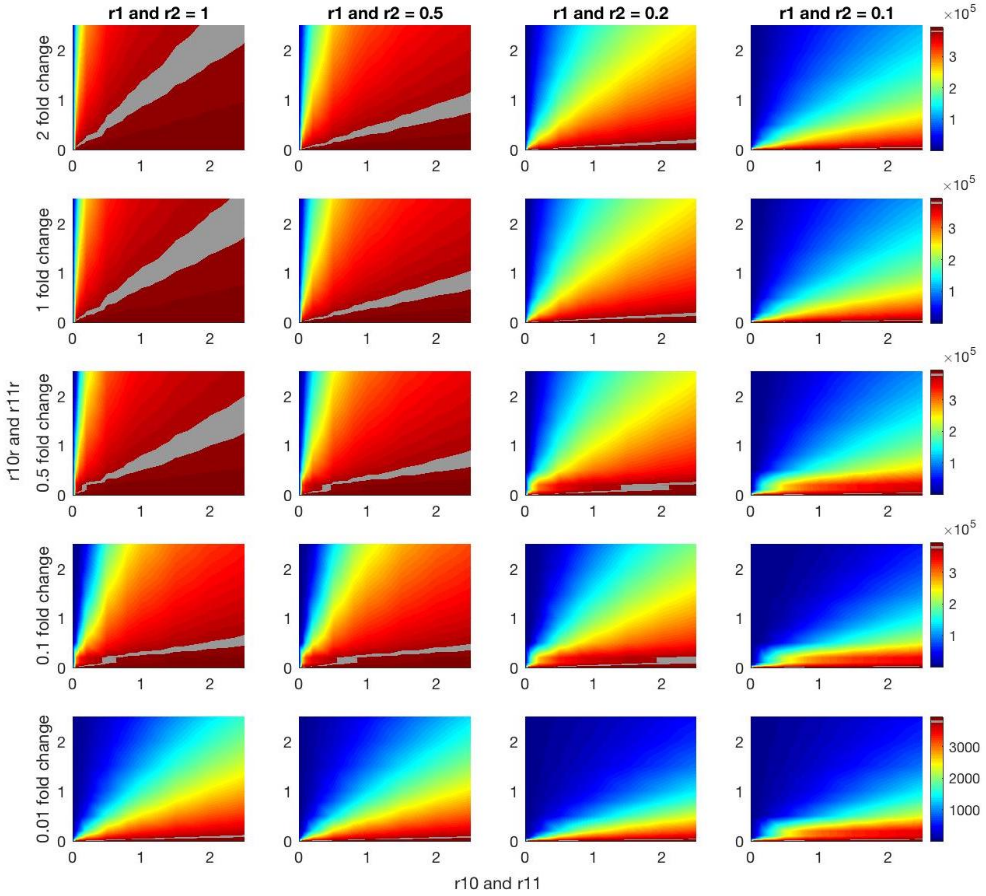

Figure 18: **PhoA yield as the area under the curve (AUC).** Heatmaps display the activity of various promoter designs for pPhoA and pPhoB, and the resulting PhoA expression, whereby the model is calibrated under varying stress response conditions due to P_i starvation, implemented as fold changes. The results are compared by calculating the yield of PhoA gene expression measured as the AUC. Each row displays the resulting system with a different fold change applied to r14, varying from 2 to 0.01 from the top to the bottom. The row with the fold change of 1 is same as the one in Figure 4. The heatmaps are ordered decreasingly from left to right according to the external P_i concentration given by the fold changes applied to the PhoR autphosphorylation reactions r1 and r2. The left most column with a fold change value of 1 is the starvation condition with $0\mu M$ external P_i. Each heatmap scans 100 simulations by applying 10 different fold change values to the promoter binding rates r10 and r11 as well as 10 different fold change values to the promoter unbinding rates r10r and r11r.

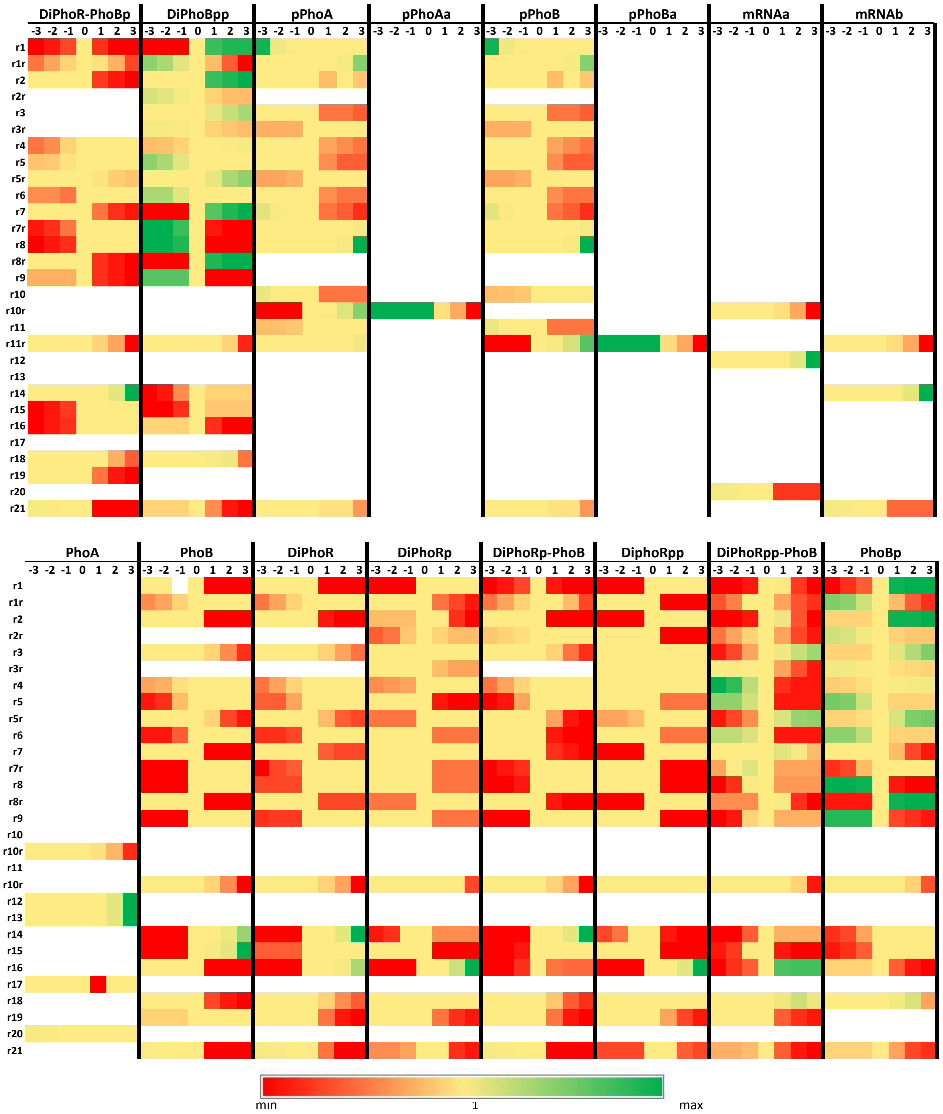

Figure 19: Heatmap displaying the results of the sensitivity analysis by considering all the model parameters. Each parameter is varied by 3 orders of magnitude higher and lower and the area under the curve (AUC) for each species is computed. The outcome is normalised with the AUC of the original model. Red represents the decreasing effect and green represents the increasing effect. The model is sensitive to a parameter when a variation in the input parameter causes a change in the model output. The amount of change quantifies the sensitivity. The sensitivity analysis results predict the system behaviour under varying conditions.

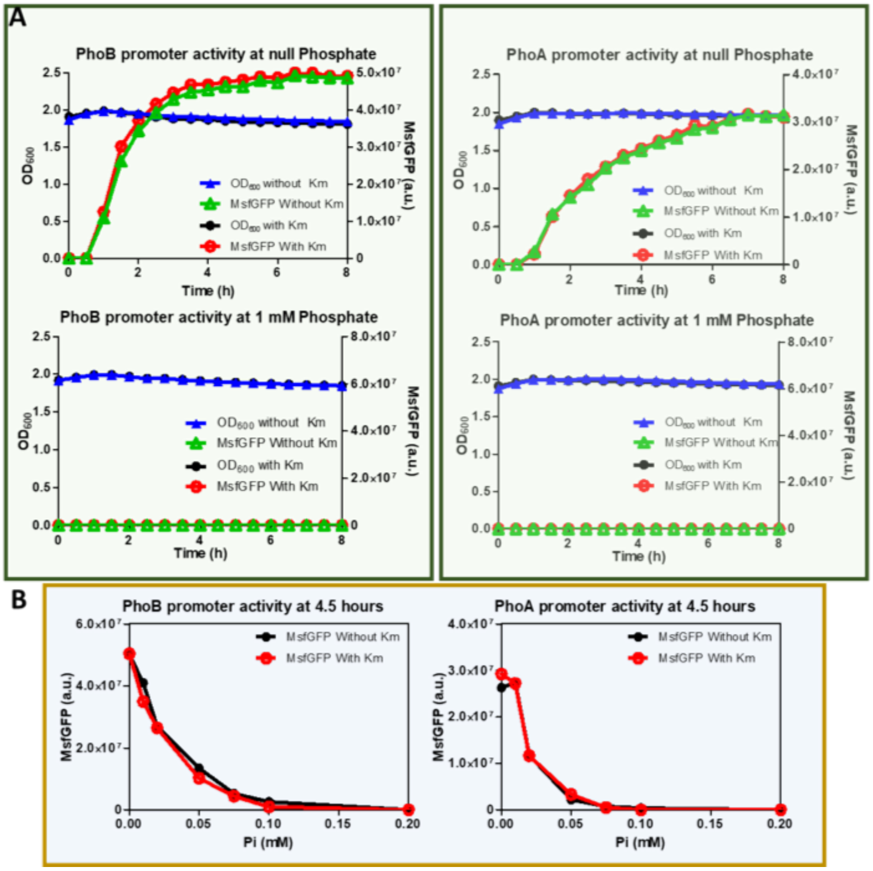

Figure 20: (A) Compared activity of the PhoA and PhoB promoters measured as MsfGFP expression in the presence or absence of $50\mu g/ml$ kanamycin (Km) and at null or $1mM$ phosphate (Pi) concentrations. (B) PhoB and PhoA activity in the presence or absence of Km after 4.5 hours at increasing Pi concentrations.
